# An integrative genomic analysis of the Longshanks selection experiment for longer limbs in mice

**DOI:** 10.7554/eLife.42014

**Published:** 2019-06-06

**Authors:** João PL Castro, Michelle N Yancoskie, Marta Marchini, Stefanie Belohlavy, Layla Hiramatsu, Marek Kučka, William H Beluch, Ronald Naumann, Isabella Skuplik, John Cobb, Nicholas H Barton, Campbell Rolian, Yingguang Frank Chan

**Affiliations:** 1 Friedrich Miescher Laboratory of the Max Planck SocietyTübingenGermany; 2 University of CalgaryCalgaryCanada; 3 Institute of Science and Technology (IST) AustriaKlosterneuburgAustria; 4 Max Planck Institute for Molecular Cell Biology and GeneticsDresdenGermany; Austrian Academy of SciencesAustria; University of MichiganUnited States

**Keywords:** selection experiment, quantitative genetics, population genetics, selective sweeps, limb development, enhancers, Mouse

## Abstract

Evolutionary studies are often limited by missing data that are critical to understanding the history of selection. Selection experiments, which reproduce rapid evolution under controlled conditions, are excellent tools to study how genomes evolve under selection. Here we present a genomic dissection of the Longshanks selection experiment, in which mice were selectively bred over 20 generations for longer tibiae relative to body mass, resulting in 13% longer tibiae in two replicates. We synthesized evolutionary theory, genome sequences and molecular genetics to understand the selection response and found that it involved both polygenic adaptation and discrete loci of major effect, with the strongest loci tending to be selected in parallel between replicates. We show that selection may favor de-repression of bone growth through inactivating two limb enhancers of an inhibitor, *Nkx3-2*. Our integrative genomic analyses thus show that it is possible to connect individual base-pair changes to the overall selection response.

## eLife digest

Humans have been making use of artificial selection for thousands of years. Much of what we eat, for example, from beef to poultry to cereals, comes from a collection of organisms with genomes that have been completely reshaped by the actions of generations of farmers and breeders. Yet, despite decades of research in evolutionary biology, it remains difficult to predict what will happen to an organism’s genes when selective pressure is applied.

Traits that at first seem simple often arise from layers upon layers of complexity. It can take hundreds if not thousands of tiny changes to many genes, plus just the right alterations to a few key ones, to have a desired effect on a single trait. Also, if you consider that often the genomes of the starting population are unknown and that many traits are under simultaneous selection in wild populations, it becomes clear why many questions remain unanswered.

Castro, Yancoskie, et al. have analyzed an on-going laboratory experiment dubbed “the Longshanks experiment” to explore how an animal’s genome changes under strong selection. Over five years, two independent populations of mice were selectively bred to have longer legs. In each generation, the mice were measured and those with the longest tibia – a bone in the shin – relative to their body mass were allowed to breed. Genetic data were also recorded. Now, Castro, Yancoskie, et al. have analyzed the genetic data up to the first 17 generations in the Longshanks experiment to find out what kind of genes may be relevant to the 13% increase in leg length seen in the mice so far.

This analysis uncovered many genes, possibly thousands, all acting in concert to increase tibia length. But the gene with the largest effect by far was a key developmental gene called *Nkx3-2*. Mutations in this gene cause a disease called spondylo-megaepiphyseal-metaphyseal dysplasia in people, which can lead to long limbs and a short trunk. Although inactivating this gene completely in mice is lethal, among the founding group of mice in the Longshanks experiment was a rare copy of *Nkx3-2*. This copy of the gene worked perfectly in all tissues with the exception of the legs, where a genetic switch that controls it was left in the “off” state. Mice inheriting this short stretch of DNA ended up having longer tibia. In effect, these mice held the winning ticket in the genetic lottery that was the Longshanks experiment.

Even in highly controlled experiments that record a great deal of information about the organisms involved, predicting how the genome will change and which genes will be involved is not a straightforward question. Finding out how the genome may change in response to selection is important because scientists can build better models to help with breeding farm animals or crops, or with predicting the consequences of climate change. As a result, experiments such as these may have broad applications in conservation, genomic medicine and agriculture.

## Introduction

Understanding how populations adapt to a changing environment is an urgent challenge of global significance. The problem is especially acute for mammal populations, which are often small and fragmented due to widespread habitat loss. Such populations often show increased inbreeding, leading to the loss of genetic diversity (Hoffmann and Sgrò, 2011). Because beneficial alleles in mammals typically come from standing genetic variation rather than new mutations like in microbes, this loss of diversity would ultimately impose a limit on the ability of small populations to adapt. Nonetheless, mammals respond readily to selection in many traits, both in nature and in the laboratory (Darwin, 1859; Gingerich, 2001; Garland and Rose, 2009; Keightley et al., 2001). In quantitative genetics, such traits are interpreted as the overall effect from a large set of loci, each with an infinitesimally small (and undetectable) effect (‘infinitesimal model’). Broadly speaking, the infinitesimal model has performed remarkably well across a wide range of selection experiments, and the model is the basis for commercial breeding (Walsh and Lynch, 2018). However, it remains unclear what type of genomic change is associated with rapid response to selection, especially in small populations where allele frequency changes can be dominated by random genetic drift.

While a large body of theory exists to describe the birth, rise and eventual fixation of adaptive variants under diverse selection scenarios (Maynard Smith and Haigh, 1974; Barton, 1995; Otto and Barton, 2001; Weissman and Barton, 2012; Crow and Kimura, 1965; Hill and Robertson, 1966), few empirical datasets capture sufficient detail on the founding conditions and selection regime to allow full reconstruction of the selection response. This is particularly problematic in nature, where historical samples, environmental measurements and replicates are often missing. Selection experiments, which reproduce rapid evolution under controlled conditions, are therefore excellent tools to understand response to selection—and by extension—adaptive evolution in nature (Garland and Rose, 2009).

Here we describe an integrative, multi-faceted investigation into an artificial selection experiment, called Longshanks, in which mice were selected for increased tibia length relative to body mass (Marchini et al., 2014). The mammalian limb is an ideal model to study the dynamics of complex traits under selection: it is both morphologically complex and functionally diverse, reflecting its adaptive value; and limb development has been studied extensively in mammals, birds and fishes as a genetic and evolutionary paradigm (Petit et al., 2017). The Longshanks selection experiment thus offers the opportunity to study selection response not only from a quantitative and population genetics perspective, but also from a developmental (Marchini and Rolian, 2018) and genomic perspective.

By design, the Longshanks experiment preserves a nearly complete archive of the phenotype (trait measurements) and genotype (via tissue samples) in the pedigree. Previously, Marchini et al. investigated how selection was able to overcome correlation between tibia length and body mass and produced independent changes in tibia length during the first 14 generations of the Longshanks experiment (Marchini et al., 2014). Importantly, that study focused on the phenotypes and inferred genetic correlations indirectly using the pedigree. The current genomic analysis was initiated when the on-going experiment reached generation 17 and extends the previous study by integrating both phenotypic *and* genetic aspects of the Longshanks experiment. By sequencing the initial and final genomes, the current analysis benefits from direct and highly resolved genetic information. Here, with essentially complete information, we wish to answer a number of important questions regarding the factors that determine and constrain rapid adaptation: Are the observed changes in gene frequency due to selection or random drift? Does rapid selection response of a complex trait proceed through innumerable loci of infinitesimally small effect, or through a few loci of large effect? What type of signature of selection may be associated with this process? Finally, when the same trait changes occur independently, do these depend on changes in the same gene(s) or the same pathways (parallelism)?

## Results

### Longshanks selection for longer tibiae

**Figure 1.**
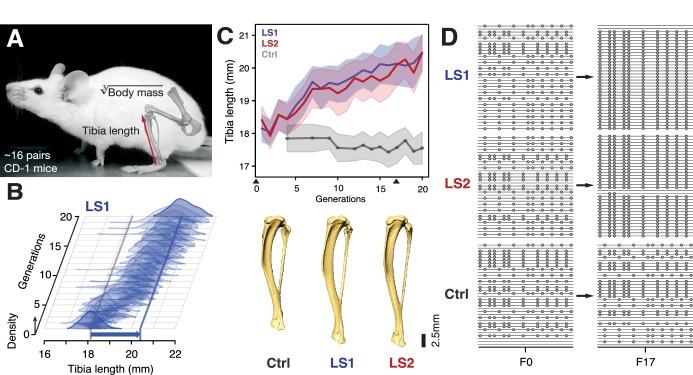
Selection for Longshanks mice produced rapid increase in tibia length. (**A and B**) Tibia length varies as a quantitative trait among outbred mice derived from the Hsd:ICR (also known as CD-1) commercial stock. Selective breeding for mice with the longest tibiae relative to body mass within families has produced a strong selection response in tibia length over 20 generations in Longshanks mice (13%, blue arrow, LS1). (**C**) Both LS1 and LS2 produced replicated rapid increase in tibia length (blue and red; line and shading show mean ±s.d.) compared to random-bred Controls (gray). Arrowheads along the x-axis mark sequenced generations F0 and F17. See Figure 1—figure supplement 1 for body mass data. Lower panel: Representative tibiae from the Ctrl, LS1 and LS2 after 20 generations of selection. (**D**) Analysis of sequence diversity data (linked variants or haplotypes: lines; variants: dots) may detect signatures of selection, such as selective sweeps (F17 in LS1 and LS2) that result from selection favoring a particular variant (dots), compared to neutral or background patterns (Ctrl). Alternatively, selection may elicit a polygenic response, which may involve minor shifts in allele frequency at many loci and therefore may leave a very different selection signature from the one shown here.

At the start of the Longshanks experiment, we established three base populations with 14 pairs each by sampling from a genetically diverse, commercial mouse stock (Hsd:ICR, also known as CD-1; derived from mixed breeding of classical laboratory mice [Yalcin et al., 2010]). In two replicate ‘Longshanks’ lines (LS1 and LS2), we bred mice by pairing 16 males and females (and excluding sibling pairs) with the longest tibia relative to the cube root of body mass for each sex. This corresponds to 15–20% of all offspring (only details essential to understanding our analysis are summarized here. See Marchini et al., 2014 for a detailed description of the breeding scheme). We kept a third Control line (Ctrl) using an identical breeding scheme, except that breeders were selected at random. In LS1 and LS2, we observed a strong and significant response to selection in tibia length (0.29 and 0.26 Haldane or standard deviations (s.d.) per generation, from a selection differential of 0.73 s.d. in LS1 and 0.62 s.d. in LS2). Over 20 generations, selection for longer relative tibia length produced increases of 5.27 and 4.81 s.d. in LS1 and LS2, respectively (or 12.7% and 13.1% in tibia length), with a modest decrease in body mass (−1.5% in LS1 and −3.7% in LS2; Student’s *t-*test, p*<*2 × 10^−4^ and p*<*1 × 10^−8^, respectively; Figure 1B and C; Figure 1—figure supplement 1; n.b. this relationship was in part biased by the F1 generation, which were fed a different diet and phenotyped three weeks later than later generations, see Marchini et al., 2014 for details). By contrast, Ctrl showed no directional change in tibia length or body mass (Figure 1C; Student’s *t*-test, p>0.05). This approximately 5 s.d. change in 20 generations is rapid compared to typical rates observed in nature (Hendry and Kinnison, 1999, but see Grant and Grant, 2002) but is in line with responses seen in selection experiments (Gingerich, 2001; Keightley et al., 2001; Falconer and Mackay, 1996; Pitchers et al., 2014).

**Figure 1—figure supplement 1.**
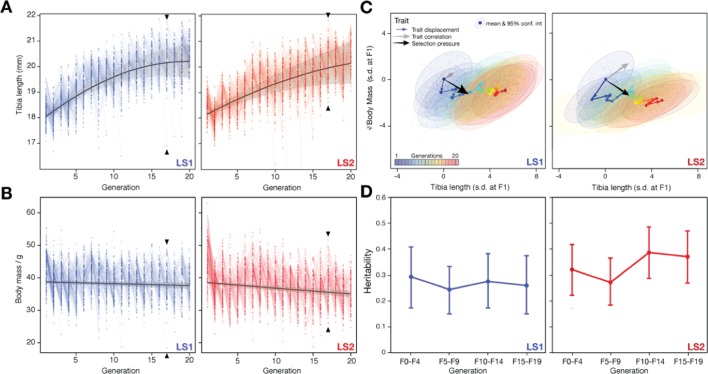
Artificial selection allowed detailed reconstruction of selection parameters. Rapid response to selection produced mice with progressively longer tibiae (**A**) and slightly lower body weight (**B**) within 20 generations. Having complete records throughout the selection experiment makes it possible to reconstruct the selection response for both phenotypes and genotypes in detail. Individuals varied in tibia length in both Longshanks lines (LS1, left; LS2, right). Lines connect parents to their offspring. The actual selection depended on the within-family and within-sex rank order of the tibia length-to-body mass (cube root) ratio (see Marchini et al., 2014 for details). The overall selection response was immediate and rapid for tibia length (**A**), suggesting a selection response that depended on standing variation among the founders (black lines show the best fitting quadratic function, with shading indicating 95% confidence interval; adjusted R^2^ = 0.61 for LS1; 0.43 for LS2). Strong selection response led to rapid increase in tibia length. In contrast, there was only minor decrease in body weight over the course of the experiment. (**C**) Trajectory in selection response shows decoupling of correlation between tibia length and body mass. Despite overall correlation between tibia length and body mass (gray arrow and major axes in confidence envelopes), cumulative trait displacement over the 20 generations (expressed in s. d. units at F1; arrows, dots and 95% confidence envelopes, color-coded according to generation) showed persistent increase in tibia length with only minor change in body mass along the general direction of selection pressure (black arrows from F1; vector length and directions based on logistic regression). This shows that the Longshanks selection experiment was successful in specifically selecting for increased tibia length while keeping relatively unchanged body mass. (**D**) Despite persistent and strong selection, heritability for the composite trait $ln\left(TB^{-0.57}\right)$ (T = tibia length in mm; B = cube-root body mass in $\sqrt[{3}]{g}$) (see *Simulating selection response: infinitesimal model with linkage* in main text) was maintained over 20 generations. Heritability was estimated by a linear mixed “animal model” in which the phenotypic variance was partitioned as *V_P_* = fixed effects + *V_A_ + V_R_*, where fixed effects were sex, age, and litter size, *V_A_* was additive genetic variance, and V_R_ was residual variance. Heritability was estimated as *h^2^* = *V_A_* /(*V_A_ + V_R_*). Each tested block used the full pedigree but only phenotypic information from individuals within the block. We tested an alternate model for each block using truncated pedigrees wherein the first generation of each block was assumed to be unrelated, but found similar results.

**Figure 1—figure supplement 2.**
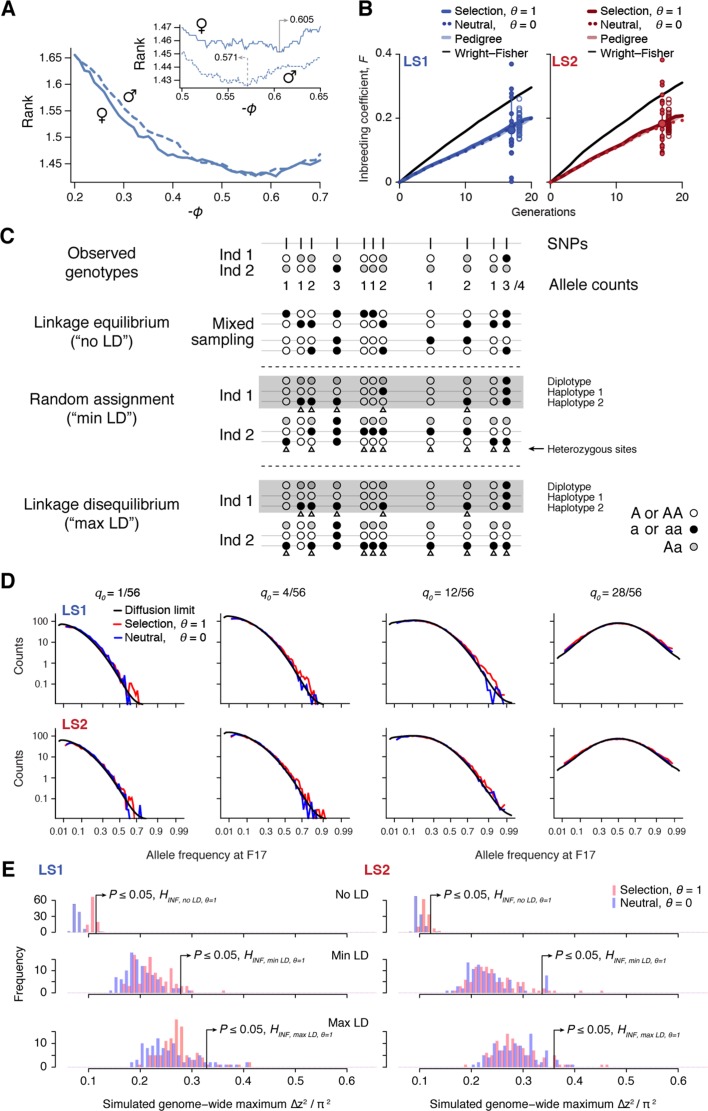
Simulating selection on pedigrees. This figure summarizes the results from our analyses to determine parameters used in the simulations. For full detail, see Appendix, section ‘*Major considerations in constructing the simulations’*. (**A**) Finding the correct ϕ value for the composite trait $ln\left(TB^{\phi }\right)$. In each simulated family, offspring are split by sex and ranked by their composite trait. Due to occasional use of back-up crosses, the average rank of actual breeders is greater than 1. We vary ϕ to find the value where actual breeders in the LS lines have the best (lowest) rank. We find ϕ = -0.571 to show the best match for males and -0.605 for females. For subsequent analyses we set ϕ to be -0.57. (**B**) Increase in inbreeding over the course of the Longshanks experiment. The lines show the change in identity between two alleles between diploid individuals, $F_{b}$, over 20 generations, as calculated from the pedigree (light shade); the average of 50 neutral simulations without selection (dotted line); or the average of 50 simulation replicates with selection intensity at $V_{s}/V_{e}$ = 0.584 (θ=1; thick, dark line). While the $F_{b}$ trajectories based on pedigree or neutral simulations are indistinguishable, inbreeding increases slightly faster under selection (thick line). The black line shows the increase in identity expected under a Wright–Fisher model with the actual population sizes; under this model, $F_{w}$ and $F_{b}$ are close to each other, and to $1-{\left(1-\frac{1}{2N_{e}}\right)}^{t}$, with $N_{e}$ equal to the harmonic mean, 24.8. The large dot (with error bar showing the interquartile range among chromosomes) at right show the actual $F_{b}$, estimated from the decline in average 2p(1−p) over 17 generations. Small filled dots show the estimates from each of the 20 chromosomes. Open dots show 40 replicate simulations, made with the same pedigree and the same selection response θ=1 and sub-sampling from the simulated chromosome according to the actual map length of each of the mouse chromosomes (Cox et al., 2009). The simulation agrees well with the observed genome-wide average. Most of the observed data from chromosomes fall within a range comparable to simulated replicates (compare large dot with open dots), with LD being the likely source of this excess variance. (**C**) Three different schemes to seed founder haplotypes. We simulate founder haplotypes that are consistent with observed genotypes (shown here as black, white and gray dots as the two homozygous and the heterozygous states) by directly sampling from founder individuals in each LS line. Under the linkage equilibrium scheme, we sample from the list of allele counts at all SNPs. This produces founder haplotypes that carry no linkage disequilibrium (‘no LD'). Under the random assignment scheme, we sample according to each individual (shown as ‘diplotypes' within the box for easy comparison). At heterozygote sites in each individual (arrowheads), we randomly assign the alleles to the two haplotypes. This produces founder haplotypes that show minimal LD that is consistent with the observed genotypes (‘min LD'). Under the ‘max LD' assignment scheme, we also sample according to each individual, except that we consistently assign its haplotypes 1 and 2 with reference (white) and alternate (black) alleles, respectively. This maximizes LD in the founder haplotypes (‘max LD'). (**D**) Simulated vs. expected allele frequency shifts. The distribution of minor allele frequencies *q_0_* at generation 17 is compared with the distribution expected with no selection (blue) or with selection (red), given a frequency of 1, 4, 12 or 28 minor alleles out of 56 founding alleles. The black line shows the diffusion limit, calculated for scaled time $\frac{17}{N_{e}}$, with $N_{e}$ estimated to be 51.7 and 48 in LS1 and LS2 respectively, from the rate of increase in F, calculated from the pedigree in panel **A** above. (**E**) Significance threshold values under varying LD from 100 simulated replicates (blue: no selection; red: observed selection response in the actual experiment, θ=1; see panel **C** on LD assignment methods). In order to account for non-independence of adjacent windows due to linkage, a distribution of genome-wide *maximum* ∆z^2^ was used to determine the significance threshold at each LD level. ∆z^2^ is the square of arcsine transformed allele frequency difference between F0 and F17; this has an expected variance of 1/2*N_e_* per generation, independent of starting frequency, and ranges from 0 to π^2^. As seen in previous panels, increasing selection pressure does produce greater shifts in ∆z^2^ despite using the same pedigree due to a relatively greater proportion of additive genetic variance $V_{s}$. However, a far greater impact on ∆z^2^ is due to changes in LD. This is because weak associations between large numbers of SNP can greatly inflate the variance of ∆z^2^. Of the three LD levels, ‘max LD' likely produced overly conservative thresholds, whereas ‘min LD' may lead to higher false positives. We have opted conservatively to use maximal LD in our analysis.

### Simulating selection response: infinitesimal model with linkage

The rapid but generally smooth increase in tibia length in Longshanks is typically interpreted as evidence for a highly dispersed genetic architecture with no individually important loci contributing to the selection response. This is classically described under quantitative genetics as the infinitesimal model. Crucially, the appropriate null hypothesis for the genomic response here should capture “polygenic adaptation” rather than a neutral model. We therefore developed a simulation that faithfully recapitulates the artificial selection experiment by integrating the trait measurements, selection regime, pedigree and genetic diversity of the Longshanks selection experiment, in order to generate an accurate expectation for the genomic response. Using the actual pedigree and trait measurements, we mapped fitness onto tibia length T and cube-root body mass B as a single composite trait $ln\left({TB}^{\phi }\right)$. We estimated ϕ from actual data as −0.57, such that the ranking of breeders closely matched the actual composite ranking used to select breeders in the selection experiment, based on T and B separately (Marchini et al., 2014) (Figure 1—figure supplement 2A). We assumed a maximally polygenic genetic architecture using an “infinitesimal model with linkage” (abbreviated here as *H_INF_*), under which the trait is controlled by very many loci, each of infinitesimally small effect (see Appendix for details). Results from simulations seeded with actual genotypes or haplotypes showed that overall, the predicted increase in inbreeding closely matched the observed data (Figure 1—figure supplement 2B). We tested models with varying selection intensity and initial linkage disequilibrium (LD), and for each, ran 100 simulated replicates to determine the significance of changes in allele frequency (Figure 1—figure supplement 2C-E). This flexible quantitative genetics framework allowed us to explore possible changes in genetic diversity over 17 generations of breeding under strong selection.

In simulations, we followed blocks of genomes as they were passed down the pedigree. In order to compare with observations, we seeded the initial genomes with single nucleotide polymorphisms (SNPs) in the same number and initial frequencies as the data. We observed much more variation between chromosomes in overall inbreeding (Figure 1—figure supplement 2B) and in the distribution of allele frequencies (Figure 2—figure supplement 1B) than expected from simulations in which the ancestral SNPs were initially in linkage equilibrium. This can be explained by linkage disequilibrium (LD) between the ancestral SNPs, which greatly increases random variation. Therefore, we based our significance threshold tests on simulations that were seeded with SNPs drawn with LD consistent with the initial haplotypes (Figure 1—figure supplement 2C and E; see Appendix).

Because our simulations assume infinitesimal effects of loci, allele frequency shifts exceeding this stringent threshold would suggest that discrete loci contribute significantly to the selection response. An excess of such loci in either a single LS replicate or in parallel would thus imply a mixed genetic architecture of a few large-effect loci amid an infinitesimal background.

### Sequencing the Longshanks mice reveals genomic signatures of selection

To detect the genomic changes in the actual Longshanks experiment, we sequenced all individuals of the founder (F0) and 17^th^ generation (F17) to an average of 2.91-fold coverage (range: 0.73–20.6×; n = 169 with <10% missing F0 individuals; Supplementary file 1). Across the three lines, we found similar levels of diversity, with an average of 6.7 million (M) segregating SNPs (approximately 0.025%, or 1 SNP per four kbp; Supplementary file 2; Figure 2—figure supplement 1A and Figure 2—figure supplement 2). We checked the founder populations to confirm negligible divergence between the three founder populations (across-line F_ST_ on the order of 1 × 10^−4^), which increased to 0.18 at F17 (Supplementary file 2). This is consistent with random sampling from an outbred breeding stock. By F17, the number of segregating SNPs dropped to around 5.8 M (Supplementary file 2). This 13% drop in diversity (0.9M SNPs genome-wide) is predicted by drift. Our simulations confirmed this and moreover, showed that selection contributed negligibly to the drop in diversity (Appendix, Figure 1—figure supplement 2B, D).

We conclude that despite the strong selection on the LS lines, there was little perturbation to genome-wide diversity. Indeed, the changes in diversity in 17 generations were remarkably similar in all three lines, despite Ctrl not having experienced selection on relative tibia length (Figure 2—figure supplement 1A). Hence, and consistent with our simulation results (Figure 1—figure supplement 2B,D), changes in global genome diversity had little power to distinguish selection from neutral drift despite the strong *phenotypic* selection response.

**Figure 2.**
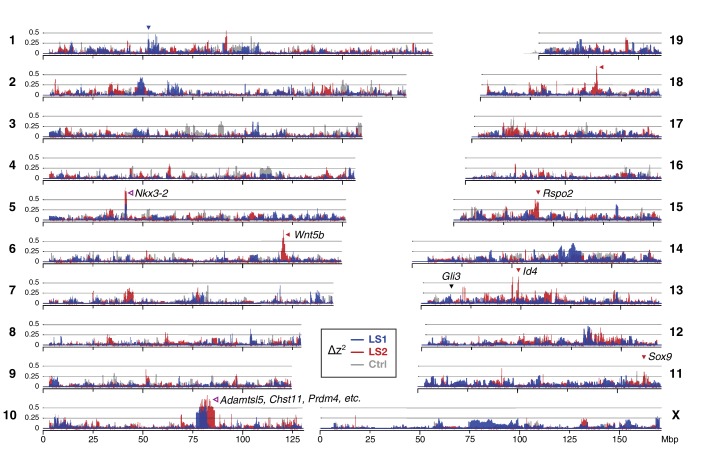
Widespread genomic response to selection for increased tibia length. Allele frequency shifts between generations F0 and F17 in LS1, LS2 and Ctrl lines are shown as ∆z^2^ profiles across the genome (plotted here as fraction of its range from 0 to π^2^). The Ctrl ∆z^2^ profile (gray) confirmed our expectation from theory and simulation that drift, inbreeding and genetic linkage could combine to generate large ∆z^2^ shifts even without selection. Nonetheless the LS1 (blue) and LS2 (red) profiles show a greater number of strong and parallel shifts than Ctrl. These selective sweeps provide support for the contribution of discrete loci to selection response (arrowheads, blue: LS1; red: LS2; purple: parallel; see also Figure 1—figure supplement 2E, Figure 2—figure supplement 2, Figure 2—figure supplement 3) beyond a polygenic background, which may explain a majority of the selection response and yet leave little discernible selection signature. Candidate genes are highlighted (Table 1). An additional *a priori* candidate limb regulator *Gli3* is indicated with a black arrowhead.

We next asked whether specific loci reveal more definitive differences between the LS replicates and Ctrl (and from infinitesimal predictions). We calculated ∆z^2^, the square of an arcsine transformed allele frequency difference between F0 and F17; this has an expected variance of 1/2*N_e_* per generation, independent of starting frequency, and ranges from 0 to π^2^. We averaged ∆z^2^ within 10 kbp windows (see Methods for details), and found 169 windows belonging to eight clusters (i.e., loci) that had significant shifts in allele frequency in LS1 and/or LS2 (corresponding to 9 and 164 clustered windows respectively at p*≤*0.05 under *H_INF, max LD_*; ∆z^2^ ≥0.33 π^2^; genome-wide ∆z^2^ = 0.02 ± 0.03 π^2^; Figure 2; Figure 1—figure supplement 2D, Figure 2—figure supplement 2, Figure 2—figure supplement 3; see Methods for details) and 8 windows in three clusters in Ctrl (genome-wide ∆z^2^ = 0.01 ± 0.02 π^2^). The eight loci in Longshanks each overlapped between 2 to 179 genes and together contained 11 candidate genes with known roles in bone, cartilage and/or limb development (e.g., *Nkx3-*2 and *Sox9*; Table 1; Figure 2—figure supplement 3, Figure 2—figure supplement 4). Four out of the eight loci contain genes with a ‘short tibia’ or ‘short limb’ knockout phenotype (Table 1; p≤0.032 from 1000 permutations, see Methods for details). Of the broader set of genes at these loci with any limb knockout phenotypes, only *fibrillin 2* (*Fbn2*) is polymorphic for SNPs coding for different amino acids, suggesting that for the majority of loci with large shifts in allele frequency, gene regulation was likely important in the selection response (Figure 2—figure supplement 4; Supplementary file 3; see Appendix for further analyses on enrichment in gene functions, protein-coding vs. *cis-*acting changes and clustering with loci affecting human height).

**Figure 2—figure supplement 1.**
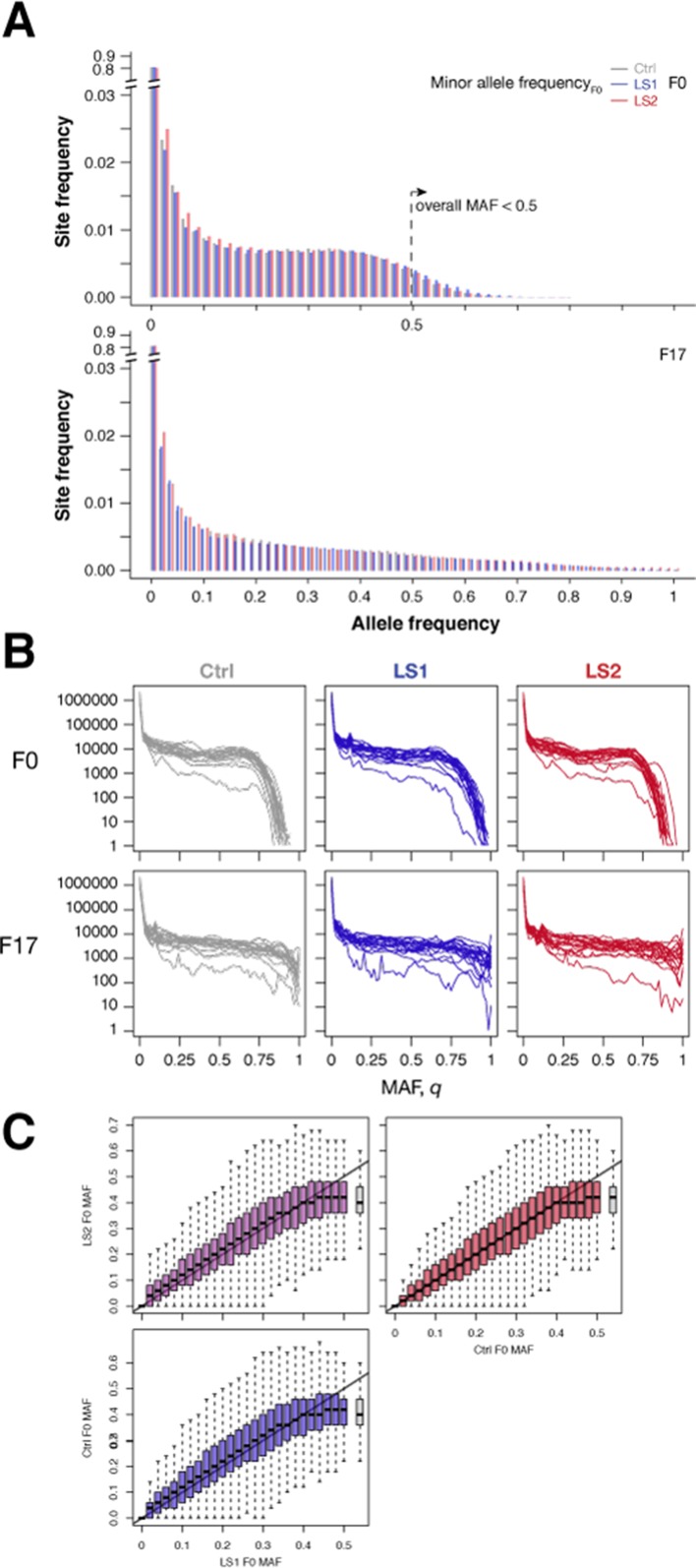
Broad similarity in molecular diversity in the founder populations for the Longshanks lines and the Control line. (**A**) Shown are the site frequency spectra from LS1, LS2 and Control lines at F0 (top; folded based on a global minor allele frequency or MAF ≤0.5) and F17 (bottom; unfolded, but tracking the same minor alleles as in F0). Overall the spectra were very similar to each other within each generation. The Control population was mostly intermediate in the decay in the rarer alleles. After 17 generations, the same alleles were generally more spread out, leading to more broadly distributed spectra. There was again little overall difference between the Longshanks and Control lines. (**B**) Variations between chromosomes (separate same-colored lines) shown in each population and generation. The unfolded site frequency spectrum is shown based on the MAF assigned as in **A**. There is substantial variation between chromosomes, which shows increased distortions in F17. (**C**) Allele frequencies between the founder populations were very similar. Joint minor allele frequencies shown as box plots in 2% bands between the Control and LS1 (blue), LS2 (red); or the two LS lines (purple). Outliers were omitted for clarity. The overall trends follow closely the parity line (gray line along the diagonal), except at frequencies very close to 0.5. Similar to the site frequency spectra in panel A, a small number of sites have a MAF above 0.5 (gray box), because of the use of an overall MAF ≤0.5 to determine minor allele status to enable comparisons across lines. Correlations between all pairwise combinations were around 0.93.

**Figure 2—figure supplement 2.**
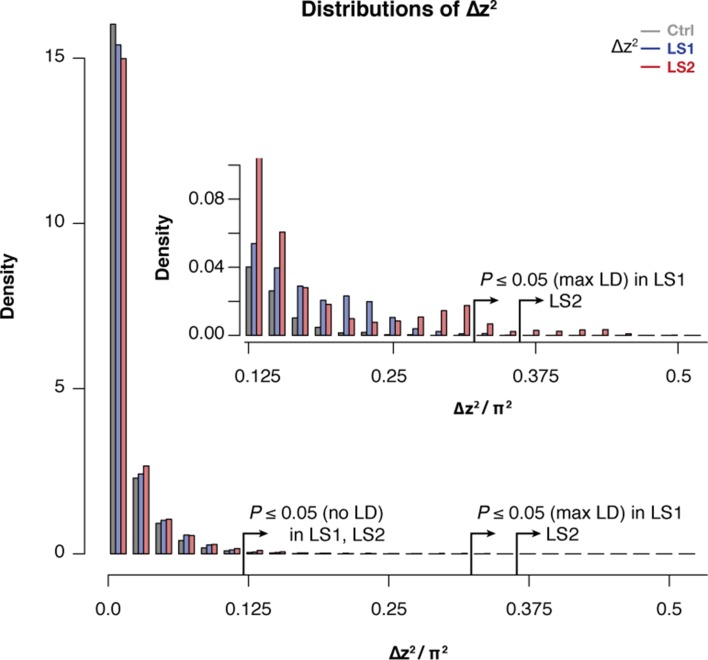
Selected lines showed more extreme values of ∆z^2^ than the Control line. Histogram of within-line ∆z^2^ values in 10 kbp windows across the genome in LS1, LS2, and Control. Overall similarity is high across all three lines, but there was an excess of large ∆z^2^ value starting from as low as <0.1 π^2^. This pattern becomes clearly distinct above the threshold value of 0.125, which corresponds to the lenient significance threshold p≤0.05 under *H_INF, no LD_* (inset). There was clearly an excess of windows in LS2 above the more stringent p≤0.05 threshold under *H_INF, max LD_*. This excess supports discrete loci contributing to selection response in LS2 that give rise to greater distortion of ∆z^2^ spectra.

**Figure 2—figure supplement 3.**
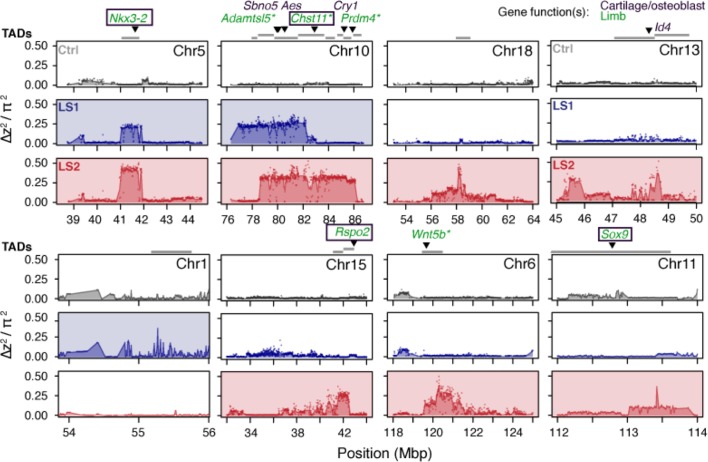
Detailed ∆z^2^ profiles at the 8 Longshanks significant loci. For each significant locus, ∆z^2^ profiles are shown for Ctrl (gray), LS1 (blue) and LS2 (red). Plots are shaded if the locus is significant in a given line. TADs within 250 kbp of the significant signals are shown as gray bars above each locus. Above the TADs are highlighted genes whose knockout phenotypes belong to the following categories: ‘abnormal tibia morphology’, ‘short limb’, ‘short tibia’, ‘abnormal cartilage morphology’, ‘abnormal osteoblast morphology’. The gene symbols are colored according to the gene function(s) in limb development (green), bone development (purple) or both (boxed). Gene symbols marked by asterisks (*) have specifically reported ‘short tibia’ or ‘short limb’ knockout phenotypes. All of the above categories show significant enrichment at the eight loci (number of genes per category: 4–7, nominal p*≤*0.03, see Appendix, section *‘Enrichment for genes with functional impact on limb development’* for details on the permutation), except ‘abnormal cartilage morphology’, with four genes and a nominal *P*-value of 0.083. No overlap was found with any gene in these categories from the three significant loci from the Ctrl line.

**Figure 2—figure supplement 4.**
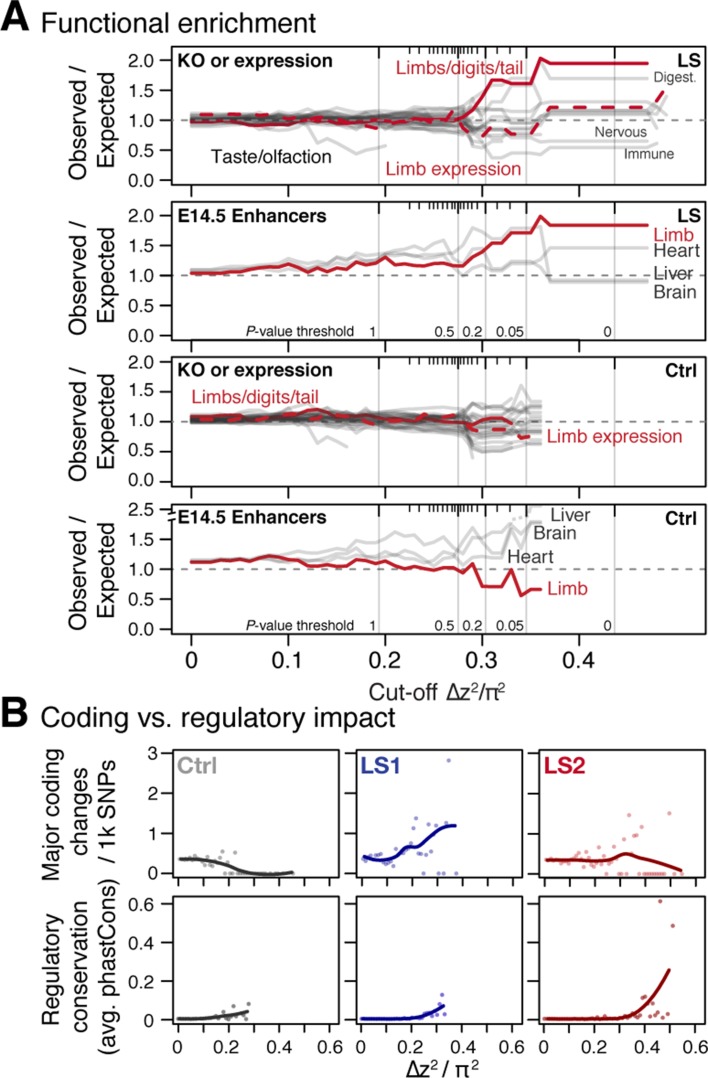
Loci associated with selection response in Longshanks lines show enrichment for limb function likely associated with *cis*-acting mechanisms. (**A**) Gene set enrichment analysis of knock-out phenotypes (KO) showed that selection response (here shown as ∆z^2^ cut-off values, see Supplementary Methods for details on cut-off values and inclusion criteria) were found among topologically associating domains (TADs) containing limb and tail developmental genes (red solid lines) or genes with limb expression (red dotted lines) in LS lines (top) but not in Ctrl (bottom). Among KO phenotypes, limb defects show the greatest excess out of 28 phenotypic categories (other gray lines, with other extreme categories labeled, the ‘normal’ category is excluded here). Among developmental enhancers for limb, heart, liver and brain tissue, we also observed an association with ∆z^2^ peaks in LS lines (top) for limb but not in Ctrl lines (bottom). The simulated significance thresholds based on *H_INF, max LD_* are also shown for reference (vertical gray lines). The data from the LS lines suggest that enrichment started to increase around the p≤0.5 threshold and remained largely stable at p≤0.05, corresponding to a cut-off of around 0.33 π^2^. (**B**) Coding vs. regulatory impact. Frequency of moderate to major coding changes (top panels, amino acid changes, frame-shifts or stop codons), or average conservation score of regulatory sequences immediately flanking SNPs (based on conservation among 60 eutherian mammals; bottom panels) were used as proxies to estimate the functional impact of coding and regulatory mutations, respectively. In LS1, major coding changes became more common at high ∆z^2^ ranges; however the number of SNPs with potentially major phenotypic consequences did not increase in LS2 and in fact seemed to decrease in Ctrl. In contrast, regulatory changes showed increased conservation associated with greater allele frequency shifts or ∆z^2^ in all three lines, except that SNPs with large shifts and strong conservation were more abundant in LS1 and LS2. Trend lines are shown with LOESS regression but statistical comparisons were performed using linear regressions.

**Table 1.** Major loci likely contributing to the selection response. These eight loci show significant allele frequency shifts in ∆z^2^ and are ordered according to their estimated selection coefficients according to Haldane (1932). Shown for each locus are the full hitchhiking spans, peak location and their size covering the core windows, the overlapping TAD and the number of genes found in it. The two top-ranked loci show shifts in parallel in both LS1 and LS2, with the remaining six showing line-specific response (LS1: 1; LS2: 5). Candidate genes found within the TAD with limb, cartilage, or bone developmental knockout phenotype functions are shown, with asterisks (*) marking those with a ‘short tibia’ knockout phenotype (see also Figure 2—figure supplement 3 and Supplementary file 3 for full table).

Rk	Chr	Span (Mbp)	Peak	Core (kbp)	TAD (kbp)	Genes	∆q				
							LS1	LS2	Ctrl	Type	Candidate genes
1	5	38.95–45.13	41.77	900	720	*3*	0.69	0.86	−0.14	Parallel	*Nkx3-2*
2	10	77.47–87.69	81.07	5360	6520	*175*	0.79	0.88	−0.04	Parallel	Sbno5, Aes, Adamtsl5*, Chst11*, Cry1, Prdm4*
3	18	53.63–63.50	58.18	220	520	*4*	0.05	0.78	−0.06	LS2-specific	-
4	13	35.59–55.21	48.65	70	2600	*22*	0.24	0.80	−0.03	LS2-specific	*Id4*
5	1	53.16–57.13	55.27	10	720	*4*	0.65	0.01	−0.23	LS1-specific	-
6	15	31.92–44.43	41.54	10	680	*3*	−0.23	0.66	0.02	LS2-specific	Rspo2*
7	6	118.65–125.25	120.30	130	1360	*12*	−0.03	0.79	−0.15	LS2-specific	Wnt5b*
8	11	111.10–115.06	113.42	10	2120	*2*	−0.14	0.66	−0.15	LS2-specific	Sox9*

Rk, Rank. Chr, Chromosome.

Core, Span of 10 kbp windows above *H_INF, max LD_ p*≤0.05 significance threshold. TAD, Merged span of topologically associating domains (TAD) overlapping the core span. TADs mark segments along a chromosome that share a common regulatory mechanism. Data from Dixon et al. (2012).

Candidate genes, Genes within the TAD span showing ‘short tibia’, ‘short limbs’, ‘abnormal osteoblast morphology’ or ‘abnormal cartilage morphology’ knockout phenotypes are listed, with * marking those with ‘short tibia’.

Taken together, two major observations stand out from our genomic survey. One, a polygenic, infinitesimal selection model with strong LD among marker SNPs performed better than moderate LD or no LD (Figure 1—figure supplement 2E); and two, we nevertheless find more discrete loci in LS1 and LS2 than in Ctrl, beyond the significance threshold set by the infinitesimal model (Figure 2; Figure 2—figure supplement 2). Thus, we conclude that although the genetic basis of the selection response in the Longshanks experiment may be largely polygenic, evidence strongly suggests discrete loci with major effect, even when each line is considered separately.

**Figure 3.**
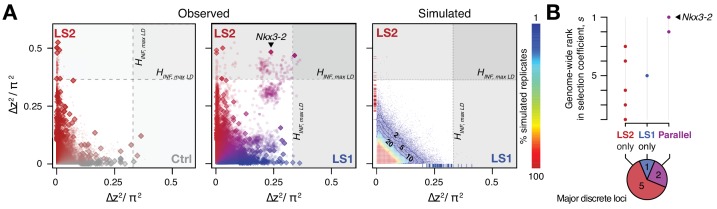
Selection response in the Longshanks lines was largely line-specific, but the strongest signals occurred in parallel. (**A**) Allele frequencies showed greater shifts in LS2 (red) than in Ctrl (gray; left panel; diamonds: peak windows; dots: other 10 kbp windows; see Figure 3—figure supplement 1 for Ctrl vs. LS1 and Appendix for details). Changes in the two lines were not correlated with each other. In contrast, there were many more parallel changes in a comparison between LS1 (blue) vs. LS2 (red; middle panel; adjacent windows appear as clusters due to hitchhiking). The overall distribution closely matches simulated results under the infinitesimal model with maximal linkage disequilibrium (*H_INF, max LD_*; right heatmap summarizes the percentage seen in 100 simulated replicates), with most of the windows showing little to no shift (red hues near 0; see also Figure 3—figure supplement 1 for an example replicate). Tick marks along the axes show genome-wide maximum ∆z^2^ shifts in each of 100 replicate simulations in LS1 (x-axis, blue) and LS2 (y-axis, red), from which we derived line-specific thresholds at the p*≤*0.05 significance level. While the frequency shifts from simulations matched the bulk of the observed data well, no simulation recovered the strong parallel shifts observed between LS1 and LS2 (compare middle to right panel, points along the diagonal). (**B**) Genome-wide ranking based on estimated selection coefficients *s* among the candidate discrete loci at p*≤*0.05 under *H_INF, max LD_*. While six out of eight total loci showed significant shifts in only LS1 or LS2, the two loci with the highest selection coefficients were likely selected in parallel in both LS1 and LS2 (also see middle panel in A).

We next tested the repeatability of the selection response at the gene/locus level using the two LS replicates. If the founding populations shared the same selectively favored variants, we may observe parallelism or co-incident selective sweeps, as long as selection could overcome random drift. Indeed, the ∆z^2^ profiles of LS1 and LS2 were more similar to each other than to Ctrl (Figure 2 and 3A; Figure 3—figure supplement 1; Pearson’s correlation in ∆z^2^ from 10 kbp windows: LS1–LS2: 0.21 vs. LS1–Ctrl: 0.06 and LS2–Ctrl: 0.05). Whereas previous genomic studies with multiple natural or artificial selection replicates focused mainly on detecting parallel loci (Burke et al., 2010; Jones et al., 2012; Chan et al., 2012; Kelly and Hughes, 2018), here we have the possibility to quantify parallelism and determine the selection value of a given locus. Six out of eight significant loci at the *H_INF, max LD_* threshold were line-specific, even though all eight selected alleles were present in the F0 generation in both lines. This prevalence of line-specific loci was consistent under different significance thresholds. However, the two remaining loci that ranked first and second by selection coefficient were parallel, both with *s* > 0.3 (Figure 3B; note that as outliers, the selection coefficient may be substantially overestimated, but their rank order should remain the same), supporting the idea that the probability of parallelism can be high among those loci with the greatest selection advantage (Orr, 2005).

Finding just two parallel loci out of 8 discrete loci may appear to be low, given the genetic similarity in the founding generation and the identical selection applied to both Longshanks replicates. However, one should bear in mind the very many genetic paths to increasing tibia length under an infinitesimal model, and that the effect of drift is expected to be very strong in these small populations. In larger populations, the shift in the balance from drift to selection should result in selection being able to favor increasingly subtle variants and thus produce a greater proportion of parallel loci. However, we expect the trend of parallelism being enriched among the top loci to hold.

In contrast to the subtle differences within each line in changes in global diversity over 17 generations (Figure 2 and Figure 2—figure supplement 2), we found the signature of parallelism to be significantly enriched in the comparison between the selected replicates (χ^2^ test, LS1–LS2: p≤1 × 10^−10^), as opposed to comparisons between each selected line and Ctrl (LS1–Ctrl: p>0.01 and LS2–Ctrl: p*>*0.2, both non-significant after correcting for multiple testing), or between simulated replicates (Figure 3—figure supplement 1; see Appendix for details). Because the parallel selected loci between LS1 and LS2 have the highest selection coefficients and parallelism is not generally expected in our populations, these loci provide the strongest evidence for the role of discrete major loci. As such, the top-ranked parallel locus is the prime candidate for molecular dissection (see Figure 4—figure supplement 1 and Appendix ‘Molecular dissection of *Gli3*’ for an additional *a priori* candidate locus with known limb function).

**Figure 3—figure supplement 1.**
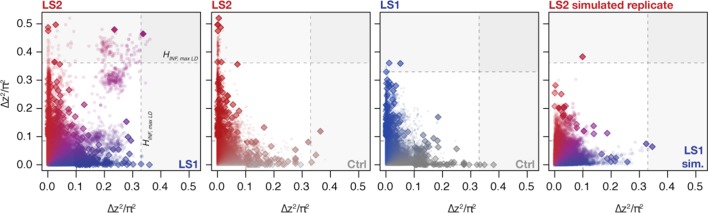
Changes in ∆z^2 ^across lines. Shown are changes in ∆z^2^ in individual 10 kbp windows (all windows: circles; peak windows: diamonds). Generally there were no clear differences in ∆z^2^ along the axes except a slight skew toward higher values in LS2. When taken as a joint LS1–LS2 comparison, however, we observed that many windows show shifts in both LS1 and LS2 (left panel; in purple). In contrast, very few windows show parallelism in Ctrl–LS2 and Ctrl–LS1 comparisons (middle two panels). The right panel shows a single selected simulated replicate (selection pressure $\frac{V_{s}}{V_{e}}=0.58$; maximum LD) found to have among the greatest extent of parallel ∆z^2^ among the replicates. The excess in parallel loci in observed results is clear both among the significant loci at p≤ 0.05 under *H_INF, max LD_* and highly significant at the more relaxed *H_INF, no LD_* threshold.

### Molecular dissection of the *Nkx3-2* locus highlights *cis*-acting changes

**Figure 4.**
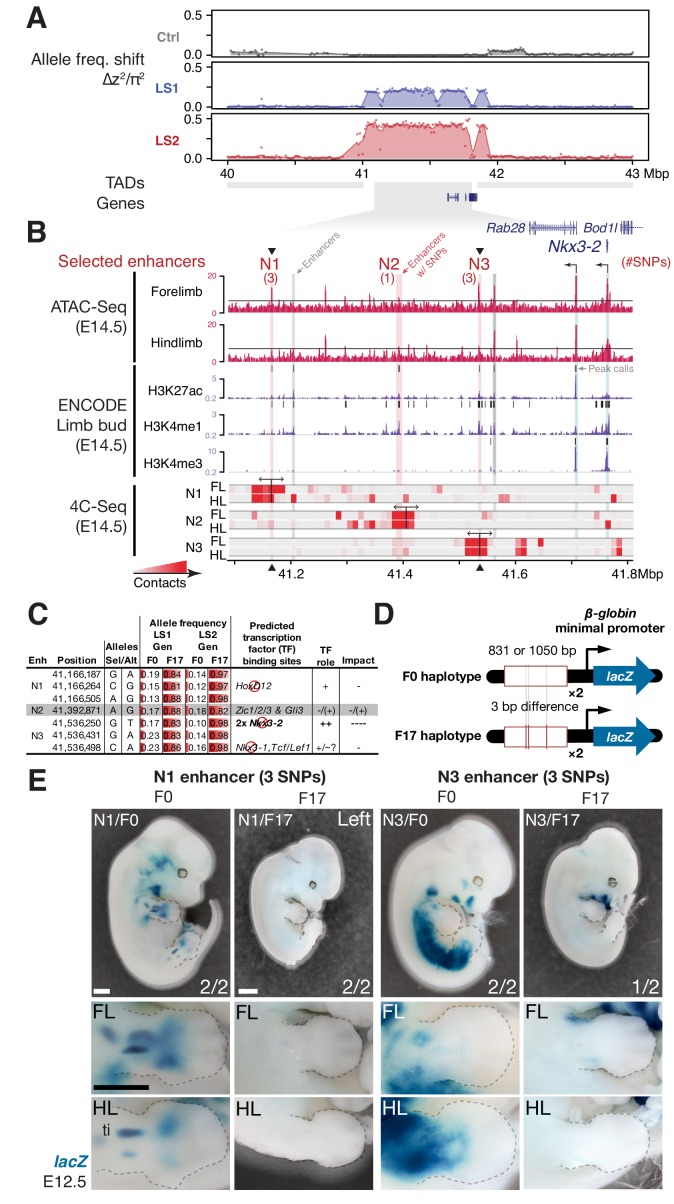
Strong parallel selection response at the bone maturation repressor *Nkx3-2* locus was associated with decreased activity of two enhancers. (**A**) ∆z^2^ in this region of chromosome five showed strong parallel differentiation spanning 1 Mbp in both Longshanks but not in the Control line. This 1 Mbp region contains three genes: *Nkx3-2*, *Rab28* and *Bod1l* (whose promoter lies outside the TAD boundary, shown as gray boxes). Although an originally rare allele in all lines, this region swept almost to fixation by generation 17 in LS2 (Figure 5—figure supplement 1A). (**B**) Chromatin profiles [ATAC-Seq, red, (Buenrostro et al., 2013); ENCODE histone modifications, purple] from E14.5 developing limb buds revealed five putative limb enhancers (gray and red shading) in the TAD, three of which contained SNPs showing significant frequency shifts. Chromosome conformation capture assays (4C-Seq) from E14.5 limb buds from the N1, N2 and N3 enhancer viewpoints (bi-directional arrows) showed significant long-range looping between the enhancers and sequences around the *Nkx3-2* promoter (heat-map from gray to red showing increasing contacts; Promoters are shown with black arrows and blue vertical shading). (**C**) Selected alleles at 7 SNPs found within the N1, N2, and N3 enhancers increased ~0.75 in frequency in both LS1 and LS2. Selected alleles at three of these sites are predicted to lead to loss (red inhibition circles) of transcription factor binding sites in the *Nkx3-2* pathway (including a SNP in N3 causing loss of two adjoining *Nkx3-2* binding sites) and thus reduce enhancer activity in N1 and N3. (**D, E**) Transient transgenic reporter assays of the N1 and N3 enhancers showed that the F0 alleles drove robust and consistent expression at centers of future cartilage condensation (N1) and broader domains of *Nkx3-2* expression (N3) in E12.5 fore- and hind limb buds (FL, HL; ti: tibia). Fractions indicate the number of embryos showing similar *lacZ* staining out of all transgenic embryos. Substituting the F17 enhancer allele (i.e., replacing three positions each in N1 and N3) led to little observable limb bud expression in both the N1/F17 and N3/F17 embryos, suggesting that selection response for longer tibia involved de-repression of bone maturation through a loss-of-function regulatory allele of *Nkx3-2* at this locus. Scale bar: 1 mm for both magnifications.

Between the two major parallel loci, we chose the locus on chromosome 5 (Chr5) at 41–42 Mbp for functional validation because it showed the strongest estimated selection coefficient, its signature of selection was clear, and crucially for functional characterization, it contains only three genes, including *Nkx3-2* (also known as *Bapx1*), a known regulator of bone maturation (Figure 2 and 4A) (Provot et al., 2006). At this locus, the pattern of variation resembles a selective sweep spanning 1 Mbp (Figure 4A). Comparison between F0 and F17 individuals revealed no recombinant in this entire region (Figure 5—figure supplement 1A, top panel), precluding fine-mapping using recombinants. We then analyzed the genes in this region to identify the likely target(s) of selection. First, we determined that no coding changes existed for either *Rab28* or *Nkx3-2,* the two genes located within the topologically associating domain (TAD, which mark chromosome segments with shared gene regulatory logic) (Dixon et al., 2012). We then performed *in situ* hybridization and detected robust expression of *Nkx3-2* and *Rab28* in the developing fore- and hind limb buds of Ctrl, LS1 and LS2 E12.5, in a domain broadly overlapping the presumptive zeugopod, the region including the tibia (Figure 4—figure supplement 2B). A third gene, *Bod1l*, straddled the TAD boundary with its promoter located in the neighboring TAD, making its regulation by sequences in the selected locus unlikely. Consistent with this, *Bod1l* showed only weak or undetectable expression in the developing limb bud (Figure 4—figure supplement 2A). We next combined ENCODE chromatin profiles and our own ATAC-Seq data to identify limb enhancers in the focal TAD. Here we found three novel enhancer candidates (N1, N2 and N3) carrying three, one and three SNPs respectively, all of which showed significant allele frequency shifts in LS1 and LS2 (Figure 4B and C; Figure 5—figure supplement 1A). Chromosome conformation capture assays showed that the N1 and N3 sequences formed long-range looping contacts with the *Nkx3-2* promoter—a hallmark of enhancers—despite as much as 600 kbp of intervening sequence (Figure 4B). We next used transgenic reporter assays to determine whether these sequences could drive expression in the limbs. Here, we were not only interested in whether the sequence encoded enhancer activity, but specifically whether the SNPs would affect the activity (Figure 4C and D). An examination of the predicted transcription factor binding sites showed that both the N1 and N3 enhancers contain multiple SNPs with consistent directional impact on the putative enhancer activity (Figure 4C). In contrast, the N2 enhancer contains only a single SNP and is predicted to have inconsistent effect on its activity. We therefore excluded the N2 enhancer from further testing. We found that the F0 alleles of the N1 and N3 enhancers (three SNPs each in about one kbp) drove robust and consistent *lacZ* expression in the developing limb buds (N1 and N3) as well as in expanded trunk domains (N3) at E12.5 (Figure 4E). In contrast, transgenic reporters carrying the selected F17 alleles of the N1 and N3 enhancers drove consistently weak, nearly undetectable *lacZ* expression (Figure 4E). Thus, switching from the F0 to the F17 enhancer alleles led to a nearly complete loss in activity (‘loss-of-function’) at developmental stage E12.5. This is consistent with the role of *Nkx3-2* as a repressor in long bone maturation (Provot et al., 2006). It should be noted that even though our selective regime favored an increase in the target phenotype (tibia length), at the molecular level we expect advantageous loss- and gain-of-function variants to be equally likely favored by selection. In fact, in an additional functional validation example at the *Gli3* locus, we found a gain-of-function enhancer variant that may have been favored at that locus (see Figure 4—figure supplement 1 and Appendix ‘Molecular dissection of *Gli3*’).

**Figure 4—figure supplement 1.**
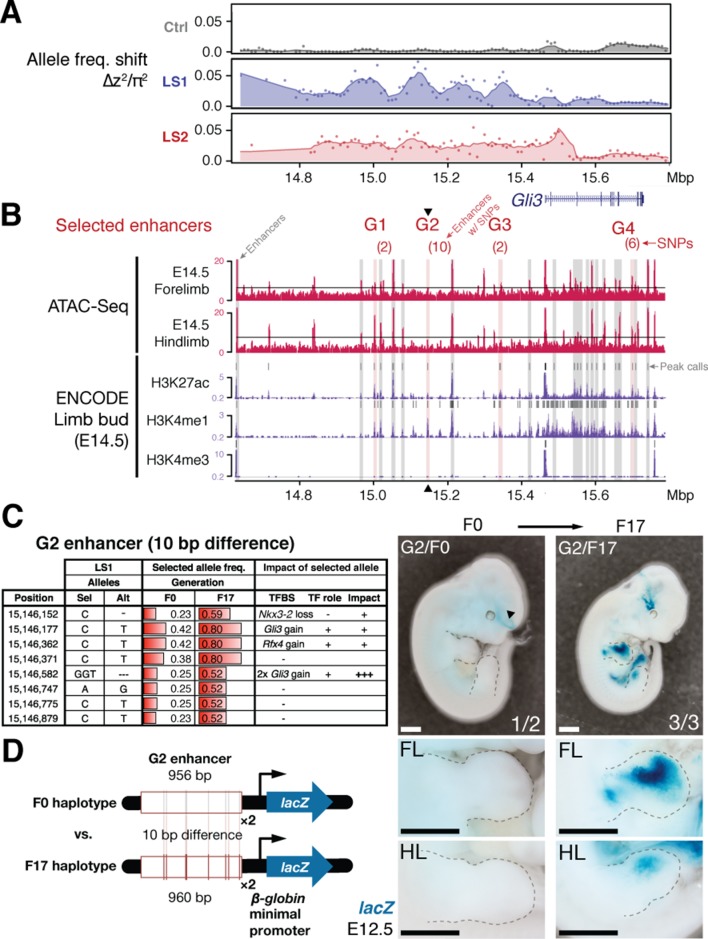
An enhancer in chromosome 13 boosts *Gli3* expression during limb bud development. (**A**) LS1 showed elevated ∆z^2^ in the intergenic region containing *Gli3*. (**B**) Putative limb enhancers (gray bars) were identified through peaks from ATAC-Seq (top) and histone modifications (bottom tracks, data from ENCODE project). Four of the enhancers contain mutations (in parentheses) with significant allele frequency shifts between F0 and F17 in LS1 (red shading). One of the enhancers located close to the peak ∆z^2^ signal (G2, arrowhead) containing 10 bp differences was chosen for transgenic reporter assay. (**C**) Analysis of individual mutations showed an average increase of 0.33 in allele frequency, with six mutated positions affecting predicted binding of transcription factors in the *Gli3* pathway (including three additional copies of *Gli3* binding sites), all of which are predicted to boost the G2 enhancer activity. (**D**) The F17 G2 enhancer variants together drove robust and consistent *lacZ* reporter gene expression at E12.5, recapitulating *Gli3* expression in the developing fore- and hind limb buds (right; see also Figure 4—figure supplement 2). Substitution of 10 positions (F0 haplotype) led to little observable expression in the limb buds (left). These G2 enhancer gain-of-function mutations (contrasting the major allele between F0 and F17) may confer an advantage under selection for increased tibia length. Scale bars: 1 mm for both magnifications.

**Figure 4—figure supplement 2.**
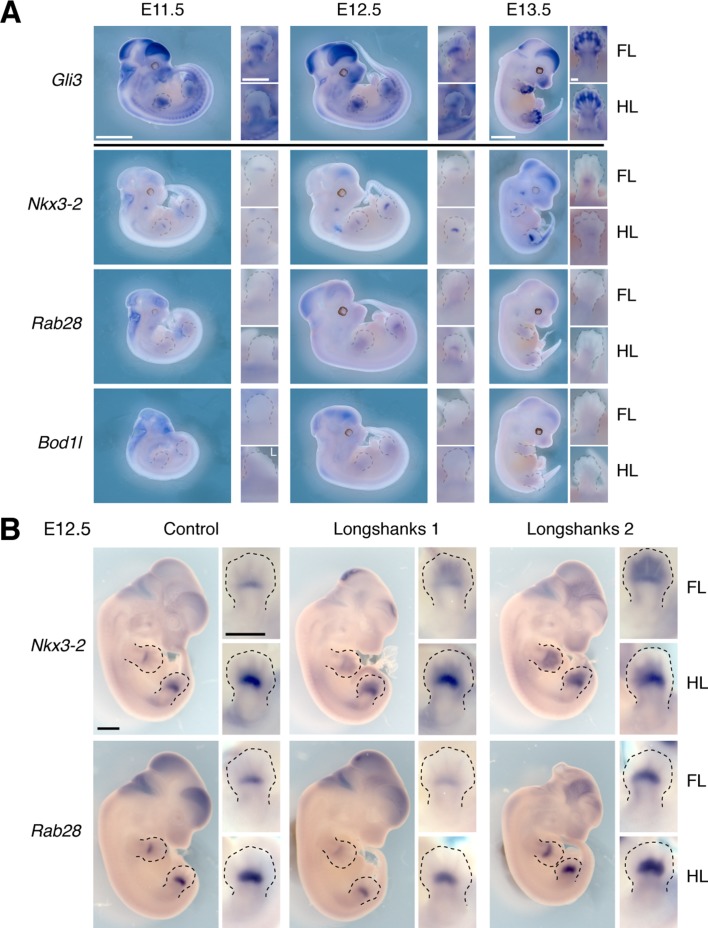
Gene expression patterns at the *Gli3* and *Nkx3-2* candidate intervals. (**A**) *Gli3* expression was determined using *in situ* hybridization. *Gli3* was robustly expressed during limb development in both developing fore- and hindlimb buds, especially in the autopod (hand/foot plate). Lower panels show expression of *Nkx3-2* and its neighboring genes *Rab28* and *Bod1l*. The stronger expression of *Nkx3-2* in the developing limb buds as well as the known role of *Nkx3-2* in bone maturation (Sivakamasundari et al., 2012) strongly argues for *Nkx3-2* being the gene underlying the selection response at the chromosome 5 locus. Scale bars: 1 mm for whole-mounts; 0.5 mm for limb buds. FL, forelimb; HL, hind limb; unless otherwise indicated by ‘L’, all images were taken from the right side. (**B**) We collected E12.5 embryos from each line and performed *in situ* hybridization to determine the sites and level of expression of *Nkx3-2* and *Rab28* in the Longshanks (right columns) and Control (left column) lines. Both genes are expressed in similar sites overall and specifically in the developing fore- and hind limb buds in the region of the presumptive zeugopods. These data indicate common sites of expression and rule out qualitative presence/absence differences in expression. Note that although the N1 enhancer pattern appear to differ from endogenous *Nkx3-2* expression (Figure 4E, details in limb buds), it matches the pattern of early *Nkx3-2* expression, detectable through the use of lineage-tracing via the combined effect of a *Nkx3-2 Cre-*driver line and the *lacZ* reporter line *Rosa26R* (see Figure 2B in Sivakamasundari et al., 2012).

At the *Nkx3-2* locus, we hypothesize that the F17 allele causes *de-repression* of bone and/or cartilage formation by reducing enhancer activity and *Nkx3-2* expression. Crucially, the F0 N1 enhancer showed activity that presages future long bone cartilage condensation in the limb (Figure 4E). That is, the observed expression pattern recalls previous results that suggest that undetected early expression of *Nkx3-2* may mark the boundaries and size of limb bone precursors, including the tibia (Sivakamasundari et al., 2012). Conversely, over-expression of *Nkx3-2* has been shown to cause shortened tibia (even loss) in mice (Bren-Mattison et al., 2011; Tribioli and Lufkin, 2006). In humans, homozygous frameshift mutations in *NKX3-2* cause the rare disorder spondylo-megaepiphyseal-metaphyseal dysplasia (SMMD; OMIM: 613330), which is characterized by short-trunk, long-limbed dwarfism and bow-leggedness (Hellemans et al., 2009). The affected bones in SMMD patients broadly correspond to the expression domains of the two novel N1 (limbs) and N3 (limbs and trunk) enhancers. Instead of wholesale loss of *Nkx3-2* expression, which would have been lethal in mice (Akazawa et al., 2000) or likely cause major defects similar to SMMD patients (Hellemans et al., 2009), our *in situ* hybridization data did not reveal qualitative differences in *Nkx3-2* expression domains between Ctrl or LS embryos (Figure 4—figure supplement 2B). Taken together, our results recapitulate the key features of a *cis*-acting mode of adaptation: *Nkx3-2* is a broadly expressed pleiotropic transcription factor that is lethal when knocked out (Akazawa et al., 2000). We found no amino acid changes between the F0 and F17 alleles that could impact protein function. Rather, selection favored changes in tissue-specific expression by modular enhancers. By combining population genetics, functional genomics and developmental genetic techniques, we were able to dissect a megabase-long locus and present data supporting the identification of up to six candidate quantitative trait nucleotides (QTNs). In mice, this represents a rare example of genetic dissection of a trait to the base-pair level.

### Linking molecular mechanisms to evolutionary consequence

**Figure 5.**
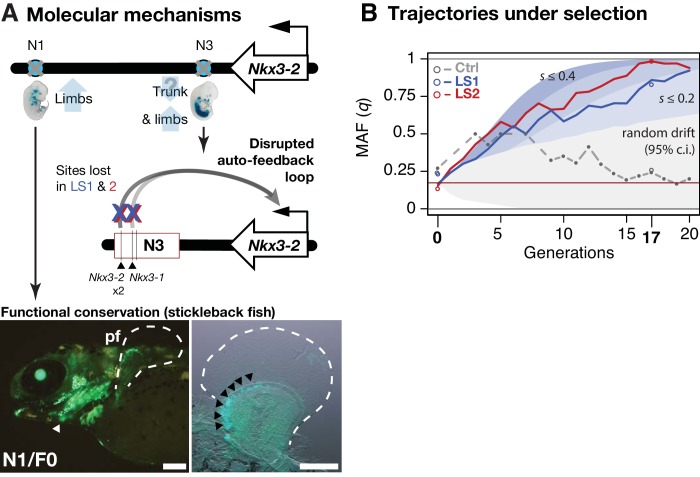
Linking base-pair changes to rapid morphological evolution. (**A**) At the *Nkx3-2* locus, we identified two long-range enhancers, N1 and N3 (circles), located 600 and 230 kbp away, respectively. During development, they drive partially overlapping expression domains in limbs (N1 and N3) and trunk (N3), which are body regions that may correlate positively (tibia length) and possibly negatively (trunk with body mass) with the Longshanks selection regime. For both enhancers, the selected F17 alleles carry loss-of-function variants (gray crosses). Two out of three SNPs in the N3 F17 enhancer are predicted to disrupt an auto-feedback loop, likely reducing *Nkx3-2* expression in the trunk and limb regions. Conversely, the enhancer function of the strong N1 F0 allele is evolutionarily conserved in fishes, demonstrated by its ability to drive consistent GFP expression (green) in the pectoral fins (pf, outlined) and branchial arches (white arrowhead, left) in transgenic stickleback embryos at 11 days post-fertilization. The N1 enhancer can recapitulate *nkx3.2* expression in distal cells specifically in the endochondral radial domain in developing fins (black arrowheads, right). Scale bar: 250 µm for both magnifications. (**B**) Allele frequency of the selected allele (minor allele at F0, q) at N3 over 20 generations (blue: LS1; red: LS2; gray broken line: Ctrl; results from N1 were nearly identical due to tight linkage). Observed frequencies from genotyped generations in the Ctrl line are marked with filled circles. Dashed lines indicate missing Ctrl generations. Open circles at generations 0 and 17 indicate allele frequencies from whole genome sequencing. The allele frequency fluctuated in Ctrl due to random drift but followed a generally linear increase in the selected lines from around 0.17 to 0.85 (LS1) and 0.98 (LS2) by generation 17. Shaded contours mark expected allelic trajectories under varying selection coefficients starting from 0.17 (red horizontal line; the average starting allele frequency between LS1 and LS2 founders). The gray shaded region marks the 95% confidence interval under random drift.

We next aimed to determine the evolutionary relevance of the *Nkx3-2* enhancer variants at the molecular and the population levels. At the strongly expressed N3/F0 ‘trunk and limb’ enhancer, we note that the SNPs in the F17 selected allele lead to disrupted *Nkx3-1* and *Nkx3-2* binding sites (Figure 4C and 5A; UNIPROBE database [Berger et al., 2008]). This suggests that the selected SNPs may disrupt an auto-feedback loop to decrease *Nkx3-2* activity in the limb bud and trunk domains (Figure 5A). Using a *GFP* transgenic reporter assay in stickleback fish embryos, we found that the mouse N1/F0 enhancer allele was capable of driving expression in the distal cells but not in the fin rays of the developing fins (Figure 5A). This pattern recapitulates fin expression of *nkx3.2* in fish, which gives rise to endochondral radials (homologous to ulna/tibia in mice) (Crotwell and Mabee, 2007). Our results suggest that strong selection may have favored the weaker N1/F17 and N3/F17 enhancer alleles in the context of the Longshanks selection regime despite the deep functional conservation of the F0 variants.

**Figure 5—figure supplement 1.**
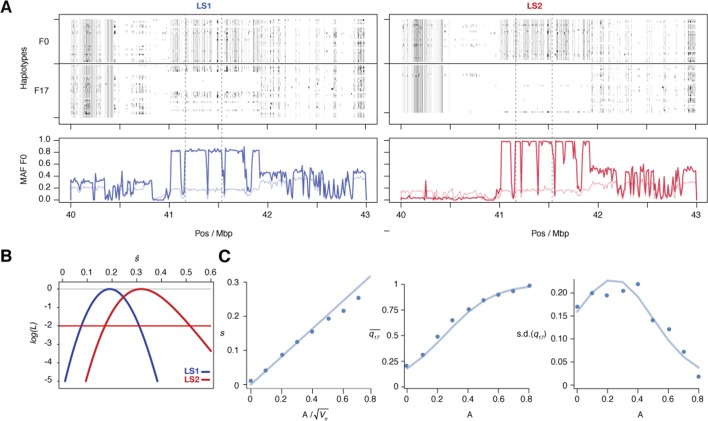
Selection at the *Nkx3-2* locus. (**A**) Raw genotypes from the F0 and F17 generations from LS1 (left) and LS2 (right) are shown, clearly indicating the area under the selective sweep. The genotype classes are shown as C57BL/6J homozygous (BL6, white), heterozygous (black) and alternate homozygous (dark gray). Lower Panel: Tracking MAF from both lines show that the originally rare F0 allele (thin line) rose to high frequency at F17 (thick lines). The plateau profile from both lines suggested that the same originally rare allele was segregating at in both founder populations and became very common by F17 in both lines (see raw genotypes). Note that in LS2 F17 the region is nearly fixed for the BL6 allele except in the bottom-most individual. (**B**) The log likelihood of the selection coefficient, *s*, for LS1 and LS2 (blue and red, respectively), based on transition probabilities for a Wright–Fisher population with the appropriate *N_e_*. The horizontal red line shows a loss of log likelihood of 2 units, which sets conventional 2-unit support limits. (**C**) Simulations of an additive allele with effect *A* on the trait; 40 replicates for each value of *A*. Left: The selection coefficient, estimated from the change in mean allele frequency, as a function of $A/\sqrt{V_{e}}$; the line shows the least-squares fit $s=0.41A/\sqrt{V_{e}}$. Middle: dots show the mean allele frequency at generation 17; the line shows the prediction from the single-locus Wright–Fisher model, given $s=0.41A/\sqrt{V_{e}}$. Right: the same, but for the standard deviation of allele frequency.

Using theory and simulations, we went beyond qualitative molecular dissection to quantitatively estimate the selection coefficient at the *Nkx3-2* locus and its contribution to the total selection response in the Longshanks mice. We retraced the selective sweep of the *Nkx3-2* N1 and N3 alleles through targeted genotyping in 1569 mice across all 20 generations. The selected allele steadily increased from around 0.17 to 0.85 in LS1 and 0.98 in LS2 but fluctuated around 0.25 in Ctrl (Figure 5B). We estimated that such a change of around 0.7 in allele frequency would correspond to a selection coefficient *s* of ~0.24 ± 0.12 at this locus (Figure 5—figure supplement 1B; see Appendix section on *‘Estimating selection coefficient of the top-ranking locus, Nkx3-2, from changes in allele frequency’*). By extending our simulation framework to allow for a major locus against an infinitesimal background, we find that the *Nkx3-2* locus would contribute 9.4% of the total selection response (limits 3.6–15.5%; see Appendix section ‘*Estimating selection coefficient*’ for details) in order to produce a shift of 0.7 in allele frequency over 17 generations. To avoid inflation stemming from estimating from outliers, we also independently estimated the contribution of the *Nkx3-2* locus using a linear mixed animal model based on the full genotyped series mentioned above (see Appendix section ‘*Estimating selection coefficient, animal model*’ for details). Using this alternative approach, we estimated that each selected allele increases tibia length by 0.36% (*N* = 1569, 95% conf. int.: 0.07–0.64%, p=0.0171). Multiplying the effect with the increase in the allele frequency suggests that the *Nkx3-2* locus alone would account for approximately 4% of the overall 12.9% increase in tibia length. This lower estimate of around 4% is nonetheless within the bounds of the estimate from simulations. Together, both approaches indicate that the *Nkx3-2* locus contributes substantially to the selection response.

## Discussion

A defining task of our time is to understand the factors that determine and constrain how small populations respond to sudden environmental changes. Here, we analyze the replicated and controlled Longshanks experiment to characterize the genomic changes that occur as small experimental populations respond to selection.

An important conclusion from the Longshanks experiment is that selection response can be steady and robust even in extremely bottlenecked populations. That is, we found that tibia length increased readily and repeatedly in response to selection even with as few as 14–16 breeding pairs per generation. The sustained response was possible because the lines were founded with enough standing variation, and generation 17 was still only a fraction of the way to the expected limit for the selection response at ~2*N_e_* generations (Robertson, 1960), estimated here to be around 90 (see legend for Figure 1—figure supplement 2B; Appendix on ‘Estimating the selection coefficient’). Although other selective breeding studies using a similar base population of mice encountered selection limits at around generation 20–25 (possibly due to countervailing selection rather than loss of genetic variance) for high voluntary wheel running behavior (Careau et al., 2013) and for nest-building behavior (Bult and Lynch, 2000), here all evidence suggests that the Longshanks mice should continue to show increases in tibia length for many more generations.

The estimated *N_e_* of 46 in the Longshanks experiment, while small, is comparable to those in natural populations like the Soay sheep (McRae et al., 2005), Darwin’s finches (Grant and Grant, 1992) or Tasmanian devils (Epstein et al., 2016) (this last study documents a rapid and parallel evolutionary response to transmissible tumors). These populations span a wide range of time in sustained bottlenecking, from the most recent in Tasmanian devils, to likely many millions of years in Darwin’s finches. Accordingly, we also expect very different dynamics during short- vs. long-term selection response: for a short bout of selection, such as the 20 generations analyzed in this study, selection response depends overwhelmingly on standing genetic variation, with little to no contribution from *de novo* mutations (Hill, 1982; Weber and Diggins, 1990). Over the long term, however, *de novo* mutations would contribute increasingly to selection response. In the Longshanks experiment, we observe a robust early response to selection (Figure 1B and Figure 1—figure supplement 1), and a gradual decrease in sequence diversity, consistent with the effect of drift (Figure 1—figure supplement 2B and Figure 2—figure supplement 1A, Supplementary file 2). There has long been broad empirical support for adaptation from standing genetic variation in nature (Jones et al., 2012; Epstein et al., 2016; Hancock et al., 2011) and breeding (Sheng et al., 2015). At least in the short-run, our result demonstrating robust selection response in the Longshanks experiment provides grounds for some optimism regarding the ability of populations to respond rapidly to changes in their environment.

By combining pedigree records with sequencing of founder individuals, our data had sufficient detail to allow precise modeling of trait response, with predicted shifts in allele frequency distribution that closely matched our results (e.g. Figure 1—figure supplement 2D). Furthermore, we functionally validated loci that showed allele frequency shifts outside the model’s predictions and found key enhancers of major effect. Connecting trait changes to allele frequency changes at specific loci has been a longstanding objective in selection experiments, with a number of notable early attempts (e.g., Keightley et al., 1996). To date, we know of only a few studies that attempt to explicitly link traits with changes in allele frequencies (Kessner and Novembre, 2015; Rice and Townsend, 2012; Chen et al., 2019; Nuzhdin et al., 1999) and none have systematically tested the underlying architecture against an infinitesimal background. Here, our results imply a mixed genetic architecture with a few discrete loci of large effect amid an infinitesimal background. It remains to be seen whether other evolve-and-resequence (E&R) studies, with different selection pressures and population parameters, may reveal similar results.

To put our finding of a mixed genetic architecture into perspective, it is worth noting that the infinitesimal model is still the most predictive model by far in practical quantitative genetics, for diverse domesticated species from cattle to crops, despite its intrinsically unrealistic assumptions (Hill et al., 2008; Lynch and Walsh, 1998; Hill and Zhang, 2012). In general, current genomic data for many traits is consistent with a very large number of loci, each with a small effect. From a practical point of view, however, the use of an infinitesimal model does not preclude the presence or indeed the importance of a few major effect loci. Rather, it simply assumes that they are rare enough to allow reasonable model fit (Walsh and Lynch, 2018, page 878). Here, we note that it is actually not clear how one might parameterize a generally applicable predictive oligogenic model with more than a single major effect locus. In this study, while we consider the most likely genetic architecture underlying selection response for tibia length to be a small number of major effect loci together with a polygenic background, we cannot reject other alternative models that could also account for the observed response, such as an effectively infinitesimal model with linkage, as well as models with a few major trait loci.

Among other classical examples of complex traits, such as height or body weight, that may have been subjected to selection, we observe a range of genetic architectures in ways often tightly connected to their population size and/or selection history. Height in humans is often cited as the classical complex trait under possible selection of unknown (and much debated) intensity (see Turchin et al., 2012; Berg and Coop, 2014; Barton et al., 2019). It shows high heritability and a highly dispersed genetic architecture (with the top-ranked locus accounting for only 0.8% of the variation explained in cosmopolitan European populations) (Weedon et al., 2007; Wood et al., 2014). In contrast, as few as 4 to 6 loci account for 83% and 50% of the variation in height in horses and dogs, respectively (Makvandi-Nejad et al., 2012; Rimbault et al., 2013). In both horses and dogs, selection has been strong and sustained, and breed-specific populations tend to be small. Interestingly, and in line with our experiment, the major allele at the *IGF1* locus stems from a standing genetic variant, despite many factors that may theoretically favor large-effect *de novo* mutations (Sutter et al., 2007). In chickens, modern breeding practice and selection from large populations yielded a highly polygenic genetic architecture for body weight, with some of the best empirical evidence for epistasis (Carlborg et al., 2006; Wahlberg et al., 2009; Rubin et al., 2010; Pettersson et al., 2013). Similarly, results from many selection experiments in *Drosophila* suggest that the genetic architecture underlying selection response may involve many genes (Jha et al., 2015; Reeves and Tautz, 2017; Orozco-terWengel et al., 2012; Turner et al., 2011). By contrast, the extreme tail of the effect size distribution (as inferred from ∆z^2^) from the Longshanks experiment appears to account for a substantial part of the selection response, presumably due to the combined effects of relatively low diversity in commercial mouse stocks and the small founding populations. But unlike these previous QTL studies or selection experiments, in which either the genetic architecture of a trait or the selection value were estimated separately, sometimes from only few parental individuals or lines, E&R studies sample a much broader pool of alleles and continually compete them against each other. Thus, our approach allowed simultaneous inference of genetic architecture and distribution of effect sizes, is more likely to be representative of the population at large, and is more akin to genome-wide association studies (GWAS), except that here we can also directly connect a trait to its selective value and capture the trajectory of any given allele.

Parallel evolution is often seen as a hallmark for detecting selection (Chan et al., 2012; Schluter et al., 2004; Chan et al., 2010; Martin and Orgogozo, 2013). We investigated the factors that contribute to parallelism in allele frequency shifts over 17 generations by contrasting the two Longshanks replicates against the Control line. However, we observed little parallelism between selected lines and Ctrl, or between simulated replicates under selection, even though the simulated haplotypes were sampled directly from actual founders. This underscores that parallelism depends on both shared selection pressure (absent in Ctrl) *and* the availability of large-effect loci that confer a substantial selection advantage (absent under the infinitesimal model; Figure 3; Figure 3—figure supplement 1). With increasing population size, selection would be better able to detect variants with more subtle effects. This would in turn lower the threshold beyond which the selective advantage of an allele would become deterministic, that is, exhibit parallelism.

Through in-depth dissection of the *Nkx3-2* locus, our data show in fine detail how the selective value of standing variants depends strongly on the selection regime: the originally common F0 variant of the N1 enhancer shows deep functional conservation and can evidently recapitulate fin *nkx3.2* expression in fishes (Figure 5A). Yet, in the Longshanks experiment selection strongly favored the weaker allele (Figure 5B). In fact, our molecular dissection of two loci show that both gain-of-function (*Gli3*) and loss-of-function (*Nkx3-2*) variants could be favored by selection (Figure 4E and 5A; Figure 4—figure supplement 1D). Through synthesis of multiple lines of evidence, our work uncovered the key role of *Nkx3-2*, which was not an obvious candidate gene like *Gli3* due to the lack of abnormal limb phenotype in *Nkx3-2* knockout mice. To our surprise, the same loss of *NKX3-2* function in human SMMD patients manifests in opposite ways in different bone types as short trunk and long limbs (Hellemans et al., 2009). This matches the expression domains of our N1 (limb) and N3 (limb and trunk) enhancers (Figure 5A). Evidently, in the absence of lethal coding mutations, the F17 haplotype was doubly beneficial at both enhancers for the limb and potentially also trunk target tissues under the novel selection regime in the Longshanks selection experiment. We estimate that these enhancer variants, along with any other tightly linked beneficial SNPs, segregate as a single locus, which in turn contributes ~10% of the overall selection response.

Despite our efforts to uncover the mechanism underlying the selective advantage of the *Nkx3-2* locus, much remains unknown. For example, it remains unclear how such a major allele could segregate in the general mouse stock (and as the reference C57BL/6J allele, no less). It could be that this allele has the same effect in the general mouse population but is conditionally neutral under non-selective breeding and simply escaped notice. However, our preliminary exploration in a panel of C57BL/6-by-DBA/2 (‘BXD’) mice suggested otherwise: mapping of tibia length or mineral density did not reveal this locus as a major QTL determining tibia length (unpublished data kindly provided by Weikuan Gu), suggesting that this allele’s effect on tibia length may depend on the genetic background. Alternatively, the broader C57BL/6 allele could be linked to a compensatory mutation that became uncoupled among the founders of the Longshanks lines. Finally, although we do observe the specific N1 and N3 SNP positions as variable across the rodent and indeed the broader mammalian lineages, further work is needed to determine their effect, if any, on limb development.

### Conclusion

Using the Longshanks selection experiment and synthesizing theory, empirical data and molecular genetics, we show that it is possible to identify some of the individual SNPs that have contributed to the response to selection on morphology. In particular, discrete, large-effect loci are revealed by their parallel response. Further work should focus on dissecting the mechanisms behind the dynamics of selective sweeps and/or polygenic adaptation by re-sequencing the entire selection pedigree, testing how the selection response depends on the genetic architecture, and the extent to which linkage places a fundamental limit on our inference of selection. Improved understanding in these areas may have broad implications for conservation, rapid adaptation to climate change and quantitative genetics in medicine, agriculture and in nature.

## Materials and methods

### Animal care and use

All experimental procedures described in this study have been approved by the applicable University institutional ethics committee for animal welfare at the University of Calgary (HSACC Protocols M08146 and AC13-0077); or local competent authority: Landesdirektion Sachsen, Germany, permit number 24–9168.11-9/2012-5.

### Reference genome assembly

All co-ordinates in the mouse genome refer to *Mus musculus* reference mm10, which is derived from GRCm38.

### Code and data availability

Sequence data have been deposited in the SRA database under accession number SRP165718 and GEO under GSE121564, GSE121565 and GSE121566. Non-sequence data have been deposited at Dryad, doi:10.5061/dryad.0q2h6tk. Analytical code and additional notes have been deposited in the following repository: https://github.com/evolgenomics/Longshanks (Evolgenomics, 2019; copy archived at https://github.com/elifesciences-publications/Longshanks). Additional raw data and code are hosted via our institute's FTP servers at http://ftp.tuebingen.mpg.de/fml/ag-chan/Longshanks/.

### Pedigree data

Tibia length and body weight phenotypes were measured as previously described (Marchini et al., 2014). A total of 1332 Control, 3054 LS1, and 3101 LS2 individuals were recorded. Five outlier individuals with a skeletal dysplasia of unknown etiology were removed from LS2 and excluded from further analysis. Missing data in LS2 were filled in with random individuals that best matched the pedigree. Trait data were analyzed to determine response to selection based on the measured traits and their rank orders based on the selection index.

### Simulations

Simulations were based on the actual pedigree and selection scheme, following one chromosome at a time. Each chromosome was represented by a set of junctions, which recorded the boundaries between genomes originating from different founder genomes; at the end, the SNP genotype was reconstructed by seeding each block of genome with the appropriate ancestral haplotype. This procedure is much more efficient than following each of the very large number of SNP markers. Crossovers were uniformly distributed, at a rate equal to the map length (Cox et al., 2009). Trait value was determined by a component due to an infinitesimal background (*V_g_*); a component determined by the sum of effects of 10^4^ evenly spaced discrete loci (*V_s_*); and a Gaussian non-genetic component (*V_e_*). The two genetic components had variance proportional to the corresponding map length, and the heritability was estimated from the observed trait values (see Appendix section ‘Major considerations’). In each generation, the actual number of male and female offspring were generated from each breeding pair, and the male and female with the largest trait value were chosen to breed.

SNP genotypes were assigned to the founder genomes with their observed frequencies. However, to reproduce the correct variability requires that we assign founder *haplotypes*. This is not straightforward, because low-coverage individual genotypes cannot be phased reliably, and heterozygotes are frequently mis-called as homozygotes. We compared three procedures, which were applied within intervals that share the same ancestry: assigning haplotypes in linkage equilibrium (LE, or ‘no LD’); assigning the two alleles at heterozygous sites in each individual to its two haplotypes at random, which minimizes linkage disequilibrium but is consistent with observed diploid genotypes (‘min LD’); and assigning alleles at heterozygous sites in each individual to the ‘reference’ and ‘alternate’ haplotype consistently within an interval, which maximizes linkage disequilibrium (‘max LD’) (Figure 1—figure supplement 2C). For details, see legend in Figure 1—figure supplement 2.

### Significance thresholds

To obtain significance thresholds, we summarized the genome-wide maximum ∆z^2^ shift for each replicate of the simulated LS1 and LS2 lines, averaged within 10 kb windows, and grouped by the selection intensity and extent of linkage disequilibrium (LD). From this distribution of genome-wide maximum ∆z^2^ we obtained the critical value for the corresponding significance threshold (typically the 95^th^ quantile or p=0.05) under each selection and LD model (Figure 3A; Figure 1—figure supplement 2E). This procedure controls for the effect of linkage and hitchhiking, line-specific pedigree structure, and selection strength.

### Sequencing, genotyping and phasing pipeline

Sequencing libraries for high-throughput sequencing were generated using TruSeq or Nextera DNA Library Prep Kits (Illumina, Inc, San Diego, USA) according to manufacturer’s recommendations or using equivalent *Tn5* transposase expressed in-house as previously described (Picelli et al., 2014). Briefly, genomic DNA was extracted from ear clips by standard Protease K digestion (New England Biolabs GmbH, Frankfurt am Main, Germany) followed by AmpureXP bead (Beckman Coulter GmbH, Krefeld, Germany) purification. Extracted high-molecular weight DNA was sheared with a Covaris S2 (Woburn, MA, USA) or ‘tagmented’ by commercial or purified *Tn5*-transposase according to manufacturer’s recommendations. Each sample was individually barcoded (single-indexed as N501 with N7XX variable barcodes; all oligonucleotides used in this study were synthesized by Integrated DNA Technologies, Coralville, Iowa, USA) and pooled for high-throughput sequencing by a HiSeq 3000 (Illumina) at the Genome Core Facility at the MPI Tübingen Campus. Sequenced data were pre-processed using a pipeline consisting of data clean-up, mapping, base-calling and analysis from software fastQC v0.10.1 (Andrews, 2016); trimmomatic v0.33 (Bolger et al., 2014); bwa v0.7.10-r789 (Li and Durbin, 2010); GATK v3.4–0-gf196186 modules BQSR, MarkDuplicates, IndelRealignment (McKenna et al., 2010; DePristo et al., 2011). Genotype calls were performed using the GATK HaplotypeCaller under the GENOTYPE_GIVEN_ALLELES mode using a set of high-quality SNP calls made available by the Wellcome Trust Sanger Centre (Mouse Genomes Project version three dbSNP v137 release [Keane et al., 2011]), after filtering for sites segregating among inbred lines that may have contributed to the original seven female and two male CD-1 founders, namely 129S1/SvImJ, AKR/J, BALB/cJ, BTBR *T^+^Itpr3 ^tf^*/J, C3H/HeJ, C57BL/6NJ, CAST/EiJ, DBA/2J, FVB/NJ, KK/HiJ, MOLF/EiJ, NOD/ShiLtJ, NZO/HlLtJ, NZW/LacJ, PWK/PhJ and WSB/EiJ based on (Yalcin et al., 2010). We consider a combined ~100x coverage sufficient to recover any of the 18 CD-1 founding haplotypes still segregating at a given locus. The raw genotypes were phased with Beagle v4.1 (Browning and Browning, 2016) based on genotype posterior likelihoods using a genetic map interpolated from the mouse reference map (Cox et al., 2009) and imputed from the same putative CD-1 source lines as the reference panel. The site frequency spectra (SFS) were evaluated to ensure genotype quality (Figure 2—figure supplement 1A).

### Population genetics summary statistics

Summary statistics of the F0 and F17 samples were calculated genome-wide (Weir–Cockerham F_ST_, π, heterozygosity, allele frequencies *p* and *q*) in adjacent 10 kbp windows or on a per site basis using VCFtools v0.1.14 (Danecek et al., 2011). The summary statistic ∆z^2^ was the squared within-line difference in arcsine square-root transformed MAF *q*; it ranges from 0 to π^2^. The resulting data were further processed by custom bash, Perl and R v3.2.0 (R Development Core Team, 2015) scripts.

### Peak loci and filtering for hitchhiking windows

Peak loci were defined by a descending rank ordering of all 10 kbp windows, and from each peak signal the windows were extended by 100 SNPs to each side, until no single SNP rising above a ∆z^2^ shift of 0.2 π^2^ was detected. A total of 810 peaks were found with a ∆z^2^ shift ≥0.2 for LS1 and LS2. Following the same procedure, we found 766 peaks in Ctrl.

### Candidate genes

To determine whether genes with related developmental roles were associated with the selected variants, the topologically associating domains (TADs) derived from mouse embryonic stem cells as defined elsewhere (Dixon et al., 2012) were re-mapped onto mm10 co-ordinates. Genes within the TAD overlapping within 500 kbp of the peak window (‘core span’) were then cross-referenced against annotated knockout phenotypes (Mouse Genome Informatics, http://www.informatics.jax.org). This broader overlap was chosen to include genes whose regulatory sequences (e.g., enhancers), but not necessarily their gene bodies, fall close to the peak window. We highlight candidate genes showing limb- and bone-related phenotypes, e.g., with altered limb bone lengths or epiphyseal growth plate morphology, as observed in Longshanks mice (Marchini and Rolian, 2018), of the following categories (along with their Mammalian Phenotype Ontology term and the number of genes): ‘abnormal tibia morphology/MP:0000558’ (212 genes), ‘short limbs/MP:0000547’ and ‘short tibia/MP:0002764’ (223 genes), ‘abnormal cartilage morphology/MP:0000163’ (321 genes), ‘abnormal osteoblast morphology/MP:0004986’ (122 genes). Note that we excluded compound mutants or those conditional mutant phenotypes involving transgenes. To determine if the overlap with these genes wassignificant, we performed 1000 permutations of the core span using bedtools v2.22.1 shuffle with the -noOverlapping option (Quinlan and Hall, 2010) and excluding ChrY, ChrM and unassembled scaffolds. We then followed the exact procedure as above to determine the number of genes in the overlapping TAD belonging to each category. We reported the quantile rank as the *P*-value, ignoring ties. To determine other genes in the region, we list all genes falling within the entire hitchhiking window (Supplementary file 3).

### Identification of putative limb enhancers

We downloaded publicly available chromatin profiles, derived from E14.5 limbs, for the histone H3 lysine-4 (K4) or lysine-27 (K27) mono-/tri-methylation or acetylation marks (H3K4me1, H3K4me3 and H3K27ac) generated by the ENCODE Consortium (Shen et al., 2012). We intersected the peak calls for the enhancer-associated marks H3K4me1 and H3K27ac and filtered out peaks overlapping promoters (H3K4me3 and promoter annotation according to the FANTOM5 Consortium [Forrest et al., 2014]).

### Enrichment analysis

To calculate enrichment through the whole range of ∆z^2^, a similar procedure was taken as in *Candidate genes* above. For knockout gene functions, genes contained in TADs within 500 kbp of peak windows were included in the analysis. We used the complete database of annotated knockout phenotypes for genes or spontaneous mutations, after removing phenotypes reported under conditional or polygenic mutants. For gene expression data, we retained all genes which have been reported as being expressed in any of the limb structures, by tracing each anatomy ontological term through its parent terms, up to the top-level groupings, e.g., ‘limb’, in the Mouse Genomic Informatics Gene Expression Database (Finger et al., 2017). For E14.5 enhancers, we used a raw 500 kbp overlap with the peak windows because enhancers, unlike genes, may not have intermediaries and may instead represent direct selection targets.

For coding mutations, we first annotated all SNPs for their putative effects using snpEff v4.0e (Cingolani et al., 2012). To accurately capture the per-site impact of coding mutations, we used per-site ∆z^2^ instead of the averaged 10 kbp window. For each population, we divided all segregating SNPs into up to 0.02 bands based on per-site ∆z^2^. We then tracked the impact of coding mutations in genes *known to be expressed in limbs*, as above. We reported the sum of all missense (‘moderate’ impact), frame-shift, stop codon gain or loss sites (‘high impact’). A linear regression was used to evaluate the relationship between ∆z^2^ and the average impact of coding SNPs (SNPs with high or moderate impact to all coding SNPs).

For regulatory mutations, we used the same bins spanning the range of ∆z^2^, but focused on the subset of SNPs falling within the ENCODE E14.5 limb enhancers. We then obtained a weighted average conservation score based on an averaged phastCons (Pollard et al., 2010) or phyloP (Siepel et al., 2005) score in ±250 bp flanking the SNP, calculated from a 60-way alignment between placental mammal genomes (downloaded from the UCSC Genome Browser [Kent et al., 2002]). We reported the average conservation score of all SNPs within the bin and fitted a linear regression on log-scale. In particular, phastCons scores range from 0 (un-conserved) to 1 (fully conserved), whereas phyloP is the $\left|{log}_{10}\right|$ of the *P-*value of the phylogenetic tree, expressed as a positive score for conservation and a negative score for lineage-specific accelerated change. We favored using phastCons for its simpler interpretation.

### Impact of coding variants

Using the same SNP effect annotations described in the section above, we checked whether any specific SNP with significant site-wise ∆z^2^ in either LS1 or LS2 cause amino acid changes or protein disruptions and are known to cause limb defects when knocked out. For each position we examined outgroup sequences using the 60-way placental mammal alignment to determine the ancestral amino acid state and whether the selected variant was consistent with purifying vs. diversifying selection. The resulting 12 genes that matched these criteria are listed in Supplementary file 4.

### Association with human height loci

We downloaded the set of 697 SNPs associated with loci for human height (Wood et al., 2014) to test if these loci cluster with the selected loci in the Longshanks lines. In order to facilitate mapping to mouse co-ordinates, each human SNP was expanded to 100 kbp centering on the SNP and converted to mm10 positions using the liftOver tool with the multiple mapping option disabled (Kent et al., 2002). We were able to assign positions in 655 out of the 697 total SNPs. Then for each of the 810 loci above the *H_INF, no LD_* threshold in the selected Longshanks lines, the minimal distance to any of the mapped human loci was determined using bedtools closest with the -d option (Quinlan and Hall, 2010). When a region actually overlapped, a distance of 0 bp was assigned. To generate a permuted set, the 810 loci were randomly shuffled across the mouse autosomes using the bedtools shuffle program with the -noOverlapping option. Then the exact same procedure as the actual data was followed to determine the closest interval. The resulting permuted intervals followed an approximately normal distribution, with observed results falling completely below the range of permuted results, that is, closer to height-associated human SNPs.

### *In situ* hybridization

Detection of specific gene transcripts were performed as previously described in Brown et al., 2005. Probes against *Nkx3-2*, *Rab28*, *Bod1l* and *Gli3* were amplified from cDNA from wildtype C57BL/6NJ mouse embryos (Supplementary file 5). Amplified fragments were cloned into pJET1.2/blunt plasmid backbones in both sense and anti-sense orientations using the CloneJET PCR Kit (Thermo Fisher Scientific, Schwerte, Germany) and confirmed by Sanger sequencing using the included forward and reverse primers. Probe plasmids have also been deposited with Addgene. *In vitro* transcription from the T7 promoter was performed using the MAXIscript T7 *in vitro* Transcription Kit (Thermo Fisher Scientific) supplemented with Digoxigenin-11-UTP (Sigma-Aldrich) (MPI Tübingen), or with T7 RNA polymerase (Promega) in the presence of DIG RNA labeling mix (Roche) (University of Calgary). Following TURBO DNase (Thermo Fisher Scientific) digestion, probes were cleaned using SigmaSpin Sequencing Reaction Clean-Up columns (Sigma-Aldrich) (MPI Tübingen), or using Illustra MicroSpin G-50 columns (GE Healthcare) (University of Calgary). During testing of probe designs, sense controls were used in parallel reactions to establish background non-specific binding.

### ATAC-seq library preparation and sequencing pipeline

ATAC-seq was performed on dissected C57BL/6NJ E14.5 forelimb and hindlimb. Nuclei preparation and tagmentation were performed as previously described in Buenrostro et al. (2013), with the following modifications. To minimize endogenous protease activity, cells were strictly limited to 5 + 5 min of collagenase A treatment at 37°C, with frequent pipetting to aid dissociation into single-cell suspensions. Following wash steps and cell lysis, 50,000 nuclei were tagmented with expressed *Tn5* transposase. Each tagmented sample was then purified by MinElute columns (Qiagen) and amplified with Q5 High-Fidelity DNA Polymerase (New England Biolabs) using a uniquely barcoded i7-index primer (N701-N7XX) and the N501 i5-index primer. PCR thermocycler programs were 72°C for 4 min, 98°C for 30 s, 6 cycles of 98°C for 10 s, 65°C for 30 s, 72°C for 1 min, and final extension at 72°C for 4 min. PCR-enriched samples were taken through a double size selection with PEG-based SPRI beads (Beckman Coulter) first with 0.5X ratio of PEG/beads to remove DNA fragments longer than 600 bp, followed by 1.8X PEG/beads ratio in order to select for Fraction A as described in Milani et al. (2016). Pooled libraries were run on the HiSeq 3000 (Illumina) at the Genome Core Facility at the MPI Tübingen Campus to obtain 150 bp paired end reads, which were aligned to mouse mm10 genome using bowtie2 v.2.1.0 (Langmead and Salzberg, 2012). Peaks were called using MACS14 v.2.1 (Zhang et al., 2008).

### Multiplexed chromosome conformation capture (4C-Seq)

Chromosome conformation capture (3C) template was prepared from pooled E14.5 liver, forelimb and hindlimb buds (n = 5–6 C57BL/6NJ embryos per replicate), with improvements to the primer extension and library amplification steps following (Sexton et al., 2012). The template was amplified with Q5 High-Fidelity Polymerase (New England Biolabs GmbH, Frankfurt am Main, Germany) using a 4C adapter-specific primer and a pool of 6 *Nkx3-2* enhancer viewpoint primers (and, in a separate experiment, a pool of 8 *Gli3* enhancer-specific viewpoint primers; Supplementary file 6). Amplified fragments were prepared for Illumina sequencing by ligation of TruSeq adapters, followed by PCR enrichment. Pooled libraries were sequenced by a HiSeq 3000 (Illumina) at the Genome Core Facility at the MPI Tübingen Campus with single-end, 150 bp reads. Sequence data were processed using a pipeline consisting of data clean-up, mapping, and analysis based upon cutadapt v1.10 (Martin, 2011); bwa v0.7.10-r789 (Li and Durbin, 2010); samtools v1.2 (Li et al., 2009); bedtools (Quinlan and Hall, 2010) and R v3.2.0 (R Development Core Team, 2015). Alignments were filtered for ENCODE blacklisted regions (ENCODE Project Consortium, 2012) and those with MAPQ scores below 30 were excluded from analysis. Filtered alignments were binned into genome-wide *BglII* fragments, normalized to Reads Per Kilobase of transcript per Million mapped reads (RPKM), and plotted and visualized in R.

### Plasmid construction

Putative limb enhancers corresponding to the F0 and F17 alleles of the *Gli3* G2 and *Nkx3-2* N1 and N3 enhancers were amplified from genomic DNA of Longshanks mice from the LS1 F0 (nine mice) and F17 (10 mice) generations and sub-cloned into pJET1.2/blunt plasmid backbone using the CloneJET PCR Kit (Thermo Fisher Scientific) and alleles were confirmed by Sanger sequencing using the included forward and reverse primers (Supplementary file 7). Each allele of each enhancer was then cloned as tandem duplicates with junction *SalI* and *XhoI* sites upstream of a β-globin minimal promoter in our reporter vector (see below). Constructs were screened for the enhancer variant using Sanger sequencing. All SNPs were further confirmed against the rest of the population through direct amplicon sequencing.

The base reporter construct pBeta-lacZ-attBx2 consists of a β-globin minimal promoter followed by a *lacZ* reporter gene derived from pRS16, with the entire reporter cassette flanked by double *attB* sites. The pBeta-lacZ-attBx2 plasmid and its full sequence have been deposited and is available at Addgene.

### Pronuclear injection of F0 and F17 enhancer-reporter constructs in mice

The reporter constructs containing the appropriate allele of each of the three enhancers were linearized with *ScaI* (or *BsaI* in the case of the N3 F0 allele due to the gain of a *ScaI* site) and purified. Microinjection into mouse zygotes was performed essentially as described (DiLeone et al., 2000).

At 12 d after the embryo transfer, the gestation was terminated and embryos were individually dissected, fixed in 4% paraformaldehyde for 45 min and stored in PBS. All manipulations were performed by R.N. or under R.N.’s supervision at the Transgenic Core Facility at the Max Planck Institute of Molecular Cell Biology and Genetics, Dresden, Germany. Yolk sacs from embryos were separately collected for genotyping and all embryos were stained for *lacZ* expression as previously described (Mortlock et al., 2003). Embryos were scored for *lacZ* staining, with positive expression assigned if the pattern was consistently observed in at least two embryos.

### Genotyping of time series at the *Nkx3-2* N3 locus

Allele-specific primers terminating on SNPs that discriminate between the F0 from the F17 N3 enhancer alleles were designed (rs33219710 and rs33600994; Supplementary file 8). The amplicons were optimized as a qPCR reaction to give allele-specific, present/absent amplifications (typically no amplification for the absent allele, otherwise average ∆Ct >10). Genotyping on the entire breeding pedigree of LS1 (n = 602), LS2 (n = 579) and Ctrl (n = 389) was performed in duplicates for each allele on a Bio-Rad CFX384 Touch instrument (Bio-Rad Laboratories GmbH, Munich, Germany) with SYBR Select Master Mix for CFX (Thermo Fisher Scientific) and the following qPCR program: 50°C for 2 min, 95°C for 2 min, 40 cycles of 95°C for 15 s, 58°C for 10 s, 72°C for 10 s. In each qPCR run we included individuals of each genotype (LS F17 selected homozygotes, heterozygotes and F0 major allele homozygotes). For the few samples with discordant results between replicates, DNA was re-extracted and re-genotyped or otherwise excluded.

### Transgenic reporter assays in stickleback fish

In sticklebacks, transgenic reporter assays were carried out using the reporter construct pBHR (Chan et al., 2010). The reporter consists of a zebrafish *heat shock protein 70* (*Hsp70*) promoter followed by an *eGFP* reporter gene, with the entire reporter cassette flanked by *tol2* transposon sequences for transposase-directed genomic integration. The *Nkx3-2* N1/F0 enhancer allele was cloned as tandem duplicates using the *NheI* and *EcoRV* restriction sites upstream of the *Hsp70* promoter. Enhancer orientation and sequence was confirmed by Sanger sequencing. Transient transgenic stickleback embryos were generated by co-microinjecting the plasmid (final concentration: 10 ng/µl) and *tol2* transposase mRNA (40 ng/µl) into freshly fertilized eggs at the one-cell stage as described in Chan et al. (2010).

## Data Availability

Sequencing data have been deposited in SRA (accession number SRP165718), GEO (accession numbers GSE121564, GSE121565 and GSE121566). Non-sequence data have been deposited at Dryad (doi:10.5061/dryad.0q2h6tk). Analytical code and additional notes have been deposited in the following repository: https://github.com/evolgenomics/Longshanks (copy archived at https://github.com/elifesciences-publications/Longshanks). Additional raw data and code are hosted via our institute's FTP servers at http://ftp.tuebingen.mpg.de/fml/ag-chan/Longshanks/. The following datasets were generated: CastroJPLYancoskieMNMarchiniMBelohlavySHiramatsuLKučkaMBeluchWHNaumannRSkuplikIOCobbJBartonNHRolianCPChanYF2019An integrative genomic analysis of the Longshanks selection experiment for longer limbs in miceNCBI Sequence Read ArchiveSRP16571810.7554/eLife.42014PMC660602431169497 CastroJPLYancoskieMNMarchiniMBelohlavySHiramatsuLKučkaMBeluchWHNaumannRSkuplikIOCobbJBartonNHRolianCPChanYF2019An integrative genomic analysis of the Longshanks selection experiment for longer limbs in miceNCBI Gene Expression OmnibusGSE12156410.7554/eLife.42014PMC660602431169497 CastroJPLYancoskieMNMarchiniMBelohlavySHiramatsuLKučkaMBeluchWHNaumannRSkuplikIOCobbJBartonNHRolianCPChanYF2019An integrative genomic analysis of the Longshanks selection experiment for longer limbs in miceNCBI Gene Expression OmnibusGSE12156510.7554/eLife.42014PMC660602431169497 CastroJPLYancoskieMNMarchiniMBelohlavySHiramatsuLKučkaMBeluchWHNaumannRSkuplikIOCobbJBartonNHRolianCPChanYF2019An integrative genomic analysis of the Longshanks selection experiment for longer limbs in miceNCBI Gene Expression OmnibusGSE12156610.7554/eLife.42014PMC660602431169497 CastroJPLYancoskieMNMarchiniMBelohlavySHiramatsuLKučkaMBeluchWHNaumannRSkuplikIOCobbJBartonNHRolianCPChanYF2019Data from: An integrative genomic analysis of the Longshanks selection experiment for longer limbs in miceDryad Digital Repository10.5061/dryad.0q2h6tkPMC660602431169497 The following previously published datasets were used: KeaneTMGoodstadtLDanecekPWhiteMAWongK2011Mouse Genomes Project version 3 dbSNP v137 releaseWellcome Sanger InstitutedbSNP v137 release ShenYYueFMcClearyDFYeZEdsallLKuanSWagnerUDixonJLeeLLobanenkovVVRenB2012A map of the cis-regulatory sequences in the mouse genomeENCODE Experiment MatrixMouse E14.5 Limb10.1038/nature11243PMC404162222763441 SmithCLBlakeJAKadinJARichardsonJEBultCJtheMouse Genome Database Group2018Mouse knockout phenotypesMouse Genome InformaticsMGI_PhenotypicAllele WoodAREskoTYangJVedantamS2014Defining the role of common variation in the genomic and biological architecture of adult human heightGIANT consortiumGWAS Anthropometric 2014 Height10.1038/ng.3097PMC425004925282103

## Additional files

Supplementary file 1. Sequencing Summary. For each line and generation, we individually barcoded all available individuals and pooled for sequencing. We aimed for a sequencing depth of around 100x coverage for 50–64 haplotypes per sample. Since the CD-1 mice were founded by an original import of 7 inbred females and two inbred males, we expect a maximum of 18 segregating haplotypes at any given locus. This sequencing design should give sufficient coverage to recover allele frequencies and possibly genotypes genome-wide. Our successful genome-wide imputation results validated this strategy.

Supplementary file 2. Pairwise F_ST_ and segregating sites (S) between populations. As expected, there is a general trend of decrease in diversity after 17 generations of breeding. Globally, there was a 13% decrease in diversity, but F17 populations still retained on average ~5.8M segregating SNPs (diagonal). There was very little population differentiation, as indicated by low F_ST_ among the three founder populations, however F_ST_ increases by at least 100-fold among lines by generation F17 (above diagonal, orange boxes). Within-line F_ST_ is intermediate in this respect, reaching about half of the differentiation observed between lines.

Supplementary file 3. Full details on the eight discrete loci. Listed here are the eight loci shown in Table 1, with additional details on the core span and the TAD span used to identify candidate genes, and a full list of genes within the full span.

Supplementary file 4. Detected protein-coding changes with large allele frequency shift in amino acids. Listed are genes carrying large frequency changing SNPs affecting amino acid residues. Highlighted cells indicate the line with greater frequency changes ≥ 0.34 (red text with shading). Other suggestive changes are also shown with red numbers in unshaded cells. The changed amino acids are marked using standard notations, with the directionality indicated as ‘purifying’ or ‘diversifying’ with respect to a 60-way protein sequence alignment with other placental mammals. The conservation score based on phastCons was calculated at the SNP position itself, ranging from 0 (no conservation) to 1 (complete conservation) among the 60 placental mammals. For each gene, reported knockout phenotypes of the ‘limbs/digits/tail’ category is reported, along with whether lethality was reported in any of the alleles, excluding compound genotypes. A summary of the mutant phenotype as reported by the Mouse Genome Informatics database is also included to highlight any systemic defects beyond the ‘limbs/digits/tail’ category (lethal phenotypes reported in bold).

Supplementary file 5. Oligonucleotides for in situ hybridization probes.

Supplementary file 6. Oligonucleotide primers for multiplexed 4C-seq of enhancer viewpoints at the *Nkx3-2* locus. The 4C-seq adapter and adapter-specific primer sequences are given in Sexton et al. (2012). N2-DS denotes its location as 18 kbp downstream of the actual N2 enhancer. All viewpoints are pointed towards *Nkx3-2* gene body ('+' strand).

Supplementary file 7. Oligonucleotide primers for amplifying the enhancers at the *Nkx3-2* locus. Each of the amplicons are tagged with *SalI* (forward) or *XhoI* (reverse) sites (underlined) for concatenation and flanked by *EcoRV* sites (underlined and bold) for insertion into the pBeta-lacZ-attBx2 reporter vector upstream of the β-globin minimal promoter.

Supplementary file 8. Oligonucleotide primers for allele-specific genotyping of the N3 enhancer. The primers were designed to target two SNPs (bold) in the N3 enhancer, rs33219710 and rs33600994.

Transparent reporting form

## Appendix 1

10.7554/eLife.42014.028 Major considerations in constructing the simulations In the Longshanks experiment, the highest-ranking male and the highest-ranking female from each family were chosen to breed with the highest-ranking mice from other families within a line (i.e., disallowing sibling matings). Thus, if we disregard non-Mendelian segregation, and the fraction of failed litters (15%), selection acts solely within families, on the measured traits. Such selection does not distort the pedigree and allows us to follow the evolution of each chromosome separately. Our simulations track the inheritance of continuous genomes by following the junctions between regions with different ancestry. In principle, we should simulate selection under the infinitesimal model by following the contributions to the trait of continuous blocks of chromosomes across the whole genome. However, this is computationally challenging, since the contributions of all the blocks defined by every recombination event have to be tracked. Instead, we follow a large number of discrete biallelic loci checking that the number is sufficiently large to approach the infinitesimal limit (Figure 1—figure supplement 2D). We made a further slight approximation by only explicitly modeling discrete loci on one chromosome at a time. We divided the breeding value of an individual into two components. The first, *V_g_*, is a contribution from a large number of unlinked loci, due to genes on all but the focal chromosome, as represented by the infinitesimal model. The values of this component amongst offspring are normally distributed around the mean of the parents, with its variance being:

$$V_{M}=(V_{A}/2)(1-\beta )(1-F_{ii}-F_{jj})$$

where: $V_{A}$ is the initial genetic variance, and $F_{ii}$, $F_{jj}$ are the probabilities of identity between distinct genes in each parent, i,j; $F_{ii}$, $F_{jj}$ are calculated from the pedigree; β is the fraction of genome on the focal chromosome. The second component, *V_s_*, is the sum of contributions from a large number, n, of discrete loci, evenly spaced along the focal chromosome (here we used 10,000), and contributing a fraction β of the initial additive variance. We choose these to have equal effects and random signs, ±α, such that initial allele frequencies $p_{0}=q_{0}=\frac{1}{2}$, and equal effects α, such that ${\beta V}_{A,0}=2\sum_{i=1}^{n}\alpha^{2}p_{i,0}q_{i,0}$. The initial population consists of 28 diploid individuals, matching the experiment, and loci have initial frequencies of 1, 4, 12 and 28 out of the diploid total of 56 alleles, in equal proportions. Inheritance is assumed to be autosomal, with no sex-linkage. This choice of equal effects approaches most closely to the infinitesimal model, for a given number of loci. The decrease in genetic variance due to random drift is measured by the inbreeding coefficient, defined as the probability of identity by descent, relative to the initial population. We distinguish the identity between two distinct genes within a diploid individual, $F_{w}$, from the probability of identity between two genes in different individuals, $F_{b}$. The overall mean identity between two genes chosen independently and at random from all 2N genes is $\bar{F}=\frac{2\left(N-1\right)F_{b}+F_{w}+1}{2N}$. The proportion of heterozygotes in the population decreases by a factor of ${1-F}_{w}$, the variance in allele frequency increases with $\bar{F}$, and the genetic diversity, E[2pq], decreases as $1-\bar{F}$. Figure 1—figure supplement 2B shows that in the absence of selection, the identity $F_{b}$ increases slower than expected under the Wright–Fisher model with the actual population sizes (compare light shaded lines with black lines). These differences are a consequence of the circular mating scheme, which was designed to slow the loss of variation. The dotted line shows the average F, estimated from the loss of heterozygosity in 50 replicate neutral simulations, each with 10^4^ loci on a chromosome of length *R*=1 Morgan. These are close to the prediction from the pedigree (light shaded lines), validating the simulations. The thick colored line in Figure 1—figure supplement 2B shows F, estimated in the same way from simulations that include truncation selection on a trait with within-family variance $V_{s}/V_{e}$ = 0.584 (a value we abbreviate as θ=1), which matches the observed selection response and parent-offspring regression. The rate of drift, as measured by the gradient in F over time, is significantly faster in simulations with selection, by 6.7% in LS1 and 9.8% in LS2 (Student’s *t*-test *P* ≤ 0.008 in LS1 and *P* ≤ 0.0005 in LS2). However, this effect of selection would not be detectable from any one replicate, since the standard deviation of the rate of drift, relative to the mean rate, is ~13% between replicates. On average, the observed loss of heterozygosity fits closely to that expected from the pedigree (large dot with error bars), though there is wide variation among chromosomes (filled dots), which is substantially higher than seen in simulations seeded with SNP at linkage equilibrium (compare filled and open dots). We then performed 100 simulations, seeding each founding generation with actual genotypes and using actual pedigrees, selection pressure or heritability parameters (within-family heritability $h^{2}$ of the fitness dimension: 0.51). A main conclusion from our modelling is that the overall allele frequencies were hardly perturbed by varying selection from random drift to even doubling the selection intensity. Upon closer examination, it became clear that under the standard “infinitesimal” model, selection could generate a weak but detectable excess of allele frequency sweeps compared to strict neutrality with no selection (Figure 1—figure supplement 2D, SNP classes 1/56 and 4/56). However, it would take many replicates (assuming no parallelism) for this excess to become statistically significant. Taken at face value, this result echoes many “evolve-and-resequence” (E&R) experiments based on diverse base populations that show only weak evidence of selective sweeps at specific loci (Burke et al., 2010; Orozco-terWengel et al., 2012). Broader patterns and analyses of parallelism On a broader scale, we also observed greater extent of parallelism globally than in the simulated results or with the empirical Ctrl line. For example, out of the 2405 and 2991 loci found above the *H_INF, no LD_* cut-off in LS1 and LS2, 398 were found in both lines (13%; χ^2^ test, N ~ 150,000 windows; χ^2^ = 2901.4, d.f. = 1, p≤1 × 10^−10^); whereas we found only 10 or seven overlaps in Ctrl–LS1 or Ctrl–LS2 comparisons, respectively. This difference between the LS1–LS2 in contrast to Ctrl–LS1 or Ctrl–LS2 is statistically significant (940 significant Ctrl loci at the *H_INF, no LD_* threshold; N ~ 150,000 windows; Ctrl–LS1: χ^2^ = 0.7; Ctrl–LS2: χ^2^ = 6.0; both p=n.s.; see also Figure 3—figure supplement 1). In fact, there was not a single window out of a total of 8.4 million windows from the 100 *H_INF, max LD_* replicates where both simulated LS1 and LS2 replicates simultaneously cleared the less stringent *H_INF, no LD_* threshold. In contrast to our earlier analysis in single LS replicates, the parallel selected loci in both LS replicates loci may provide the strongest evidence yet to reject the infinitesimal model. Heritability estimate by an animal model We estimated heritability using linear mixed effect “animal models” with maximum likelihood (Figure 1—figure supplement 1D) in the R package MCMCglmm v2.5 (Hadfield, 2010; following guide by de Villemereuil, 2019). Because the animal model makes inference of the parameter estimates to the base population, to compare heritability as it changed over time we estimated heritability in blocks of 5 generations F0–4, 5–9, 10–14, and 15–19, separately for each selected line. In testing each block, we used the full pedigree to build the relationship matrix but only phenotypes from the individuals in those generations. As an alternative, we tested each block with a truncated pedigree, in which the first generation of each block is treated as unrelated (i.e., the base population). The two methods produced similar results. In all analyses, we standardized the composite trait $\ln\;({TB}^{-0.57})$ (T= tibia length in mm; B = cube-root body mass in $g^{1/3}$; see *Simulating selection response: infinitesimal model with linkage* in main text) within each generation and line to account for fuctuations in mean and variance (Careau et al., 2013). The phenotypic variance was partitioned as *V*_P_ = fixed effects + *V*_A_ + *V*_R_, where fixed effects were sex, age, and litter size, *V*_A_ was additive genetic variance, and *V*_R_ was residual variance. Heritability was estimated as *h*^2^ = *V*_A_ /(*V*_A_ + *V*_R_). Enrichment for genes with functional impact on limb development To determine what types of molecular changes may have mediated the selection response, we performed a gene set enrichment analysis. We asked if the outlier loci found in the Longshanks lines were enriched for genes affecting limb development (as indicated by their knockout phenotypes) and found increasingly significant enrichment as the allele frequency shift ∆z^2^ cut-off became increasingly stringent (Figure 2—figure supplement 4A). The ‘limb/digital/tail’ category of affected anatomical systems in the Mouse Genomic Informatics Gene Expression Database (Finger et al., 2017) showed the greatest excess of observed-to-expected ratio out of all 28 phenotype categories (the excluded ‘normal’ category also showed no enrichment). In contrast, genes showing knock-out phenotypes in most other categories did not show similar enrichment as ∆z^2^ became more stringent (Figure 2—figure supplement 4A). For genes expressed in limb tissue, there was a similar, but weaker increase, with the enrichment only appearing at higher ∆z^2^ cut-off. We did not observe similar enrichment using data and thresholds derived from Ctrl (Figure 2—figure supplement 4A, lower panels). To investigate the impact on regulatory sequences, we obtained 21,211 limb enhancers predicted by ENCODE chromatin profile at a stage immediately preceding bone formation (Theiler Stage 23, at approximately embryonic day E14.5) (Shen et al., 2012). We found likewise an enrichment throughout the range of significance cut-offs (Figure 2—figure supplement 4A). Again, there was no similar enrichment in Ctrl. Clustering with loci associated with human height Since tibia lengths directly affect human height, we tested if an association exists between loci controlling human height (Wood et al., 2014) and a set of 810 loci at the p*≤*0.05 significance level under *H_INF, no LD_* described here. After remapping the human loci to their orthologous mouse positions (n = 655 out of 697 total height loci; data from the GIANT Consortium), we detected significant clustering with the 810 peak loci (mean pairwise distance to remapped height loci: 1.41 Mbp vs. mean 1.69 Mbp from 1000 permutations of shuffled peak loci, range: 1.45–1.93 Mbp; n = 655 height loci and 810 peak loci; p*<*0.001, permutations). We interpret this clustering to suggest that a shared and conserved genetic program exist between human height and tibia length and/or body mass. Genome-wide analysis of the role of coding vs. *cis*-acting changes in response to selection We examined the potential functional impact of coding or regulatory changes as a function of ∆z^2^ in all three lines. For coding changes, we tracked the functional consequences of coding SNPs of moderate to high impact (missense mutations, gain or loss of stop codons, or frame-shifts). Whereas we found only mixed evidence of increased coding changes as ∆z^2^ increased in the LS lines, there was a depletion of coding changes in Ctrl line as ∆z^2^ increased, possibly due to purifying or background selection (Figure 2—figure supplement 4B; linear regression, LS1: p*≤*0.015, slope >0; LS2: p=0.62, n.s., slope ≈ 0; Ctrl: p*≤*5.72 × 10^−9^, slope <0). For regulatory changes, we used sequence conservation in limb enhancers overlapping a SNP as a proxy for functional impact. In contrast to the situation for coding changes, where the correlations differed between LS1 and LS2, the potential impact of regulatory changes increased significantly as a function of ∆z^2^ in both LS lines (Figure 2—figure supplement 4B): within limb enhancers, SNP-flanking sequences became increasingly conserved at highly differentiated SNPs (phastCons conservation score, ranging from 0 to 1 for unconserved to completely conserved positions; linear regression, log-scale, p*<*1.05 × 10^−9^ for both, slopes > 0). This relationship also exists for the Ctrl line, albeit principally from lower ∆z^2^ and conservation values (p*<*0.8 × 10^−3^, slope >0; Figure 2—figure supplement 4B). Taken together, our enrichment analysis suggests that while both coding and regulatory changes were selected in the Longshanks experiment, the overall selection response may depend more consistently on *cis-*regulatory changes, especially for developmental regulators involved in limb, bone and/or cartilage development (Table 1; Supplementary file 3; c.f. Supplementary file 4 for coding changes). This is a key prediction of the ‘*cis-*regulatory hypothesis’, especially in its original scope on morphological traits (Carroll, 2008). Genes with amino acid changes of potentially major impact We have further identified 12 candidate genes with likely functional impact on limb development due to specific amino acid changes showing large frequency shifts (albeit only one, *Fbn2*, cleared the stringent p≤0.05 *H_INF__, max LD_* threshold; six in LS1, nine in LS2, of which three were shared; Supplementary file 4). Consistent with strong selection for tibia development, all 12 genes show limb or tail phenotypes when knocked out, e.g., ‘short limbs’ for the collagen gene *Col27a1* knockout. Most of these genes encode for structural cellular components, e.g., myosin, fibrillin and collagen (*Myo10; Fbn2*; and *Col27a1* respectively), with *Fuz* (fuzzy planar cell polarity protein) being the only classical developmental regulator gene. All but one of these genes have also been shown to have widespread pleiotropic effects with broad expression domains, and their knockouts were often lethal (eight out of 12) and/or exhibit defects in additional organ systems (11 out of 12). Based on this observation, we anticipate that the phenotypic impact of these selected coding missense SNPs (n.b. not knockout) would not be restricted to tibia or bone development. Molecular dissection of *Gli3*, a candidate limb regulator, reveal gain-of-function *cis*-acting changes We anticipated that genes related to major limb patterning, like *Gli3*, may contribute to the selection response ( Mo et al., 1997; Nakamura et al., 2015). We thus performed an in-depth molecular dissection of *Gli3*, an important early limb developmental regulator on chromosome 13 (Chr13; Figure 4—figure supplement 1A). This locus showed a substantial shift in minor allele frequency of up to 0.42 in LS1 (∆*q*, 98^th^ quantile genome-wide, but below the *H_INF, max LD_* threshold to qualify as a discrete major locus). We performed functional validation of *Gli3*, given its limb function (Büscher et al., 1997) and considering that *Gli3* could be among the many minor loci in the polygenic background contributing to the selection response in LS1. At the *Gli3* locus we could only find conservative amino acid changes (D1090E and I1326V) that are unlikely to impact protein function. Because the signal in LS1 was stronger in the 5’ flanking intergenic region, we examined the *Gli3 cis*-regulatory topologically associating domain (TADs, which mark chromosome segments with shared gene regulatory logic) (Dixon et al., 2012) and identified putative enhancers using chromatin modification marks from the ENCODE project and our own ATAC-Seq data (Figure 4—figure supplement 1B) (Buenrostro et al., 2013; Shen et al., 2012). Four putative enhancers carried SNPs with large allele frequency changes. Among them, an upstream putative enhancer G2 (956 bp) carried 6 SNPs along with two 1- and 3 bp insertion/deletion (‘indel’) with putative functional impact due to predicted gain or loss in transcription factor binding sites (Figure 4—figure supplement 1C). We tested the G2 putative enhancer in a transgenic reporter assay by placing its sequence as a tandem duplicate upstream of a *lacZ* reporter gene (see Methods for details). We found that only the F17 LS1 allele was able to drive consistent *lacZ* expression in the developing limb buds (Figure 4—figure supplement 1D). Importantly, this enhancer was active not only in the shaft of the limb bud but also in the anterior hand/foot plate, a major domain of *Gli3* expression and function (Figure 4—figure supplement 1A). Furthermore, substitution of the enhancer sequence with the F0 allele (10 differences out of 956 or 960 bp) abolished *lacZ* expression (Figure 4—figure supplement 1D). This showed that 10 or fewer changes within this novel enhancer sequence were sufficient to convert the inactive F0 allele into an active limb enhancer corresponding to the selected F17 allele (‘gain-of-function’), suggesting that a standing genetic variant of the F17 allele may have been selectively favored because it drove stronger expression of *Gli3*, a gene essential for tibia development (Akiyama et al., 2015, but see Koziel et al., 2005). Estimating the selection coefficient of the top-ranking locus, *Nkx3-2*, from changes in allele frequency The significant locus on Chr5 containing *Nkx3-2* shows strong changes in SNP frequency in both LS1 and LS2. Here, we estimate the strength of selection on this locus, and the corresponding effect on the selected trait. We approximate by assuming two alternative alleles, and find the selection coefficient implied by the observed parallel changes in allele frequency; we then set bounds on this estimate that take account of random drift. Finally, we use simulations that condition on the known pedigree to estimate the effect on the trait required to cause the observed strong frequency changes; these show that linked selection has little effect on the single-locus estimates. We see strong and parallel changes in allele frequency at multiple steps. There are 14 non-overlapping 10kb windows that have a mean square change in arc-sin transformed allele frequency of $\bar{\Delta z^{2}}\;>\;2$ in both LS1 and LS2, spanning a 260 kbp region and including 807 SNP. SNP frequencies are tightly clustered, corresponding to two alternative haplotypes (Figure 5 and Figure 5—figure supplement 1A). The initial (untransformed) allele frequencies average *q*_0_ = 0.18, 0.17 in LS1, LS2, respectively, and the final frequencies average *q*_17_ = 0.84, 0.98, respectively (also see Figure 5—figure supplement 1A, lower panel). These frequencies depend on the arbitrary threshold for which windows to include. However, this makes little difference, relative to the wide bounds on our estimates. Under constant selection, $log\frac{q}{p}$ changes linearly with time, at a rate equal to the selection coefficient, s. Therefore, a naive estimate of selection is given by $\hat{s}=\frac{1}{T}log\left[\frac{q_{17}}{p_{17}}\frac{p_{0}}{q_{0}}\right]$ (Haldane, 1932) thus, $\hat{s}$ = 0.19, 0.32 for *q* in LS1, LS2, and averages 0.26. Here, males and females with longest tibia are chosen to breed; the strength of selection on an additive allele depends on the fraction selected and the within-family trait variance. The former is kept constant, and there is little loss of variance due to drift (F~0.17). Thus, assuming constant selection is reasonable (Figure 5B), unless there is strong dominance. To set bounds on this estimate, we must account for random drift. The predicted loss of diversity over 17 generations, based on the pedigree, is F=0.173, 0.175 for LS1, LS2, which corresponds to an effective size $N_{e}=44.9,44.4$, respectively (note that due to differences in estimation methodology, this $N_{e}$ differs slightly from that mentioned in Figure 1—figure supplement 2 but is largely consistent). Therefore, we calculate the matrix of transition probabilities for a Wright–Fisher population with 2N rounded to 90, 89 copies for LS1, LS2, over a range of selection,s. This yields the probability that the number of copies would change from the rounded values of 16/90 to 75/90 in LS1, and from 14/89 to 87/89 in LS2—that is, the likelihood of s, given the observed changes in allele frequency, and the known $N_{e}$. There is no significant loss of likelihood by assuming the same selection in both lines; overall, $\hat{s}$ = 0.24 (limits 0.13–0.36; Figure 5—figure supplement 1B). Estimating the selection coefficient, accounting for linked loci The estimates above using the simple approach do not account for selection on linked loci, and do not give the effect on the composite trait. We therefore simulated conditional on the pedigree and on the actual selection regime, as described above, but including an additive allele with effect *A* at the candidate locus on Chr5. The genetic variance associated with the unlinked infinitesimal background, and across Chr5, were reduced in proportion, to keep the overall heritability the same as before $V_{a}/\left(V_{a}+V_{e}\right)=$ 0.539. The selection coefficient inferred from the simulated changes in allele frequency was approximately proportional to the effect on the trait, with best fit $s=0.41A/\sqrt{V_{e}}$ (Figure 5—figure supplement 1C, left). Assuming this relationship, we can compare the mean and standard deviation of allele frequency from simulations with linked selection, with that predicted by the single locus Wright–Fisher model (points vs. line in Figure 5—figure supplement 1C, middle and right). These agree well, showing that linked selection does not appreciably change the distribution of allele frequencies at a single locus. This is consistent with Figure 1—figure supplement 2D, which shows that linked selection only inflates the tail of the allele frequency distribution, an effect that would not be detectable at a single locus. Combining our estimates of the selection coefficient with the relation $s=0.41A/\sqrt{V_{e}}$, we estimate that the locus on Chr5 has effect $\hat{A}=0.59\sqrt{V_{e}}$, with 2-unit support limits $0.32\sqrt{V_{e}}$ to $0.87\sqrt{V_{e}}$. This single locus is responsible for ~9.4% of the total selection response (limits 3.6–15.5%). This analysis does not allow for the inflation of effect that might arise from multiple testing. This is hard to estimate, because it depends on the distribution of effects across the genome, and also on the excess variation in estimates due to LD in the founder population. However, we note that if the effect of this locus is large enough that it would certainly be detected in this study, then there is no estimation bias from this source. We also assume that there are two haplotypes, each with a definite effect. There might in fact be heterogeneity in the effects of each haplotype, for two reasons. First, this region might have had heterogeneous effects in the founder population, with multiple alleles at multiple causal loci. Second, as recombination breaks up the founder genomes, blocks of genome would become associated with different backgrounds. To the extent that genetic variation is spread evenly over an infinitesimal background, this latter effect is accounted for by our simulations, and has little consequence. However, we have not tested whether the data might be explained by more than two alleles, possibly at more than one discrete locus. Testing such complex models would be challenging, and we do not believe that such test would have much power. However, the estimates of selection made here should be regarded as effective values that may reflect a more complex reality. Estimating the contribution of the *Nkx3-2* locus using an animal model We used a linear mixed ‘animal model’ to estimate the effect of the enhancer N3 (of the major locus in *Nkx3-2*) on the composite selected trait $ln\left({TB}^{-0.57}\right)$, see Section 'Simulating selection response: infinitesimal model with linkage' and Figure 1—figure supplement 2A. The model was:

$$V_{p}\;=\;fixed\;effects\;+\;V_{A}\;+\;V_{R}$$

where: fixed effects = sex, generation, litter size (i.e., number of siblings in family), genotype at N3 (0, 1, or 2 copies of F17 allele), and replicate line *V*_A_ = additive genetic variance *V*_R_ = residual variance We found a small but significant effect of the genotype at enhancer N3 on the composite trait (mean effect = 0.36%; 95% CI: 0.069–0.64%; p=0.017). Given the same body mass B, the mean effect corresponds to 0.36% increase in tibia length per copy of the F17 allele, or ~1% of the variance in tibia length at generation F01. The observed increase of this allele from ~0.18 to 0.91, averaged over the two lines, implies that it accounts for ~4% of the total selection response. This is within the confidence limits in the main text, based on the change in SNP frequency (3.6–15.5%) and note that the latter may be biased upwards by ascertainment. However, the exact effect of the allele is difficult to pinpoint in any given generation or population due the nature of the composite trait and change in variance in the composite trait over generations.
