# Supplementary material for: An integrative genomic analysis of the Longshanks selection experiment for longer limbs in mice
Source: eLife. 2019 Jun 6;8:e42014. doi: 10.7554/eLife.42014 (PMC6606024; doi:10.7554/eLife.42014)
Supplement: Transparent reporting form [file elife-42014-transrepform.pdf]

eLIFE
1st Floor
24 Hills Road
Cambridge CB2 1JP, UK
P 01223 855340
W elifesciences.org
T @elife

eLife's transparent reporting form

We encourage authors to provide detailed information *within their submission* to facilitate the interpretation and replication of experiments. Authors can upload supporting documentation to indicate the use of appropriate reporting guidelines for health-related research (see EQUATOR Network), life science research (see the BioSharing Information Resource), or the ARRIVE guidelines for reporting work involving animal research. Where applicable, authors should refer to any relevant reporting standards documents in this form.

If you have any questions, please consult our Journal Policies and/or contact us: editorial@elifesciences.org.

Sample-size estimation

- You should state whether an appropriate sample size was computed when the study was being designed
- You should state the statistical method of sample size computation and any required assumptions
- If no explicit power analysis was used, you should describe how you decided what sample (replicate) size (number) to use

Please outline where this information can be found within the submission (e.g., sections or figure legends), or explain why this information doesn't apply to your submission:

Sample sizes are mentioned in:
L.161. L. 1175 (Methods, 4C-Seq), 1223 (*Nkx3-2* genotyping); 760, 763 (Clustering analysis).

No explicit power analysis was used in the Longshanks selection experiment. The number of two replicate lines plus Control line (three total) and the population size of the lines were guided by a combination of husbandry considerations and comparable size and scale per replicate to previously published selection experiments (see High Runner lines from the Garland Lab, reviewed in T. Garland, M. R. Rose, *Experimental evolution: concepts, methods, and applications of selection experiments*, 2009).

For 4C-Seq and ATAC-Seq samples, these were determined by referencing ENCODE standards and other similar published experiments. In such cases we were able to determine statistical significance within the samples themselves by comparing against background signals in the rest of the genome.

Replicates

- You should report how often each experiment was performed
- You should include a definition of biological versus technical replication
- The data obtained should be provided and sufficient information should be provided to indicate the number of independent biological and/or technical replicates
- If you encountered any outliers, you should describe how these were handled
- Criteria for exclusion/inclusion of data should be clearly stated

eLife Sciences Publications, Ltd is a limited liability non-profit non-stock corporation incorporated in the State of Delaware, USA, with company number 5030732, and is registered in the UK with company number FC030576 and branch number BR015634 at the address 1st Floor, 24 Hills Road, Cambridge CB2 1JP | August 2014

- High-throughput sequence data should be uploaded before submission, with a private link for reviewers provided (these are available from both GEO and ArrayExpress)

Please outline where this information can be found within the submission (e.g., sections or figure legends), or explain why this information doesn't apply to your submission:

For the pedigree simulation under the "infinitesimal model with linkage", the number of loci modelled were tested to check that it converges to the infinitesimal limit (Supplemental notes; Supplementary Methods; Fig. 1 – figure supplement 2). The population parameters were estimated from the actual experiment (Main text: L. 129-133, 137; Supplemental notes; Fig. 1 – figure supplement 2, Fig. 3, Fig. 3 – figure supplement 2). The number of simulation replicates (Main text: L. 138 – 140, n = 100) or permutations (clustering with human height loci) were chosen to be high enough to generate reasonable background distributions and were examined to confirm consistency or normality.

Statistical reporting

- Statistical analysis methods should be described and justified
- Raw data should be presented in figures whenever informative to do so (typically when N per group is less than 10)
- For each experiment, you should identify the statistical tests used, exact values of N, definitions of center, methods of multiple test correction, and dispersion and precision measures (e.g., mean, median, SD, SEM, confidence intervals; and, for the major substantive results, a measure of effect size (e.g., Pearson's r, Cohen's d)
- Report exact p-values wherever possible alongside the summary statistics and 95% confidence intervals. These should be reported for all key questions and not only when the p-value is less than 0.05.

Please outline where this information can be found within the submission (e.g., sections or figure legends), or explain why this information doesn't apply to your submission:

In general we match and within the confines of readability, try to convey as much details as possible regarding our statistical analyses. Extensive details on the statistical treatments and summary statistics are reported (for example, see section "Longshanks selection for longer tibiae", L. 84 – 104, p. 4 – 5). Details of samples, or distributions, and methods to estimate or achieve the estimates are given in Figs. 1, 3, 5B. Statistical significance, tests, and sample sizes are given in: L. 98, 102, 197 – 198, 204, 278 – 279 and in the Appendix, L. 762 – 763, 769 – 776, 782 – 785, 940; Material and Methods L. 1002 - 1009.

(For large datasets, or papers with a very large number of statistical tests, you may upload a single table file with tests, Ns, etc., with reference to sections in the manuscript.)

Group allocation

- Indicate how samples were allocated into experimental groups (in the case of clinical studies, please specify allocation to treatment method); if randomization was used, please also state if restricted randomization was applied
- Indicate if masking was used during group allocation, data collection and/or data analysis

Please outline where this information can be found within the submission (e.g., sections or figure legends), or explain why this information doesn't apply to your submission:

In this experiment, the different replicate lines make for a natural grouping of samples into their own groups. For the sequencing analysis we also grouped individuals based on their generation number. Otherwise, no explicit grouping or sample masking was used.

Additional data files ("source data")

- We encourage you to upload relevant additional data files, such as numerical data that are represented as a graph in a figure, or as a summary table
- Where provided, these should be in the most useful format, and they can be uploaded as "Source data" files linked to a main figure or table
- Include model definition files including the full list of parameters used
- Include code used for data analysis (e.g., R, MatLab)
- Avoid stating that data files are "available upon request"

Please indicate the figures or tables for which source data files have been provided:

The raw and processed sequence data are available at the following repositories under accession numbers:<br>SRA: SRP165718<br><br>GEO: GSE121564 (4C-Seq), GSE121565 (ATAC-Seq) and GSE121566 (Superseries).<br><br>Non-sequence data have been deposited at Dryad, doi:10.5061/dryad.0q2h6tk.<br><br>Original scripts and analytical code are being continuously deposited to our laboratory's Github repository:<br>https://github.com/evolgenomics/Longshanks
