# Supplementary material for: An integrative genomic analysis of the Longshanks selection experiment for longer limbs in mice
Source: eLife. 2019 Jun 6;8:e42014. doi: 10.7554/eLife.42014 (PMC6606024; doi:10.7554/eLife.42014)
Supplement: Peer review history

# Decision letter

**DOI:** 10.7554/eLife.42014.048

**Affiliations:** Austrian Academy of SciencesAustria; New York UniversityUnited States; University of KansasUnited States

In the interests of transparency, eLife includes the editorial decision letter and accompanying author responses. A lightly edited version of the letter sent to the authors after peer review is shown, indicating the most substantive concerns; minor comments are not usually included.

Thank you for submitting your article "An integrative genomic analysis of the Longshanks selection experiment for longer limbs in mice" for consideration by *eLife*. Your article has been reviewed by Patricia Wittkopp as the Senior Editor, a Reviewing Editor, and three reviewers. The following individuals involved in review of your submission have agreed to reveal their identity: Matthew V Rockman (Reviewer #2); John Kelly (Reviewer #3).

The reviewers have discussed the reviews with one another and the Reviewing Editor has drafted this decision to help you prepare a revised submission.

Summary:

This paper uses a combination of theoretical and empirical population genetics, functional genomics, and developmental genetics to elucidate the molecular basis for the response in a long-term selection experiment for mice with longer limbs. The authors demonstrate the contribution of major loci (one of which, Nkx3-2, is identified) as well as a polygenic background, providing one of the most detailed analyses of a selection response to date.

Essential revisions:

Standard *eLife* practice is to provide a condensed decision letter instead of individual reviews, but because the reviews in this case are very well written and in perfect agreement (not to mention positive!), doing so would be a disservice to the authors. Thus, we attach the full reviews and simply list the essential revisions here.

First, it must be made clearer which data are new, and which were previously published (presumably in Marchini et al., 2014).

Second, although the polygenic analysis is an important part of this paper, we noted that no formal comparison with a simpler QTL model is included. How much of the selection response was due to each? Either add some analysis along these (perhaps test how adding more loci improves cross-replicate prediction?), or tone down the language.

*Reviewer #1:*

The manuscript by Castro et al., dissects the genomic signatures underlying the phenotypic response to artificial selection in the Longshank experiment. The authors find that the response to selection is a composite of subtle allele frequency changes across the whole genome (polygenic response) and major allele frequency changes in some individual loci. Interestingly, they find that only two of such "major effect" loci are found in both replicates, with rest of them being unique to each replicate line. They focus on one of the major effect loci, and dissect it down to three SNPs that result in a de-repression of bone growth.

This is a well written and easy to read manuscript, with a very interesting and rigorously explored dataset. I particularly like the way the authors push the story from a "signature of selection" perspective to explore the relative importance of polygenic vs major-effect-loci underlying rapid response to selection. The array of assays and datasets used across the paper were very well integrated. In particular, I find that the use of TADs to define candidate regions added more meaning to such definition than the traditionally-used LD blocks. I think the evolutionary community, in general, will be interested in this manuscript.

1) This study is based on the selection experiment by Marchini et al., (2014). However, in the text it is never clear which data are new and which are from Marchini, 2014 (e.g. there they explore mice up to the thirteenth generation). The only hint is in the Introduction. The way the text is currently written suggests the Lonshank experiment is a new contribution of this manuscript.

2) The authors claim that an infinitesimal model with linkage "best fits the observed data" (subsection “Sequencing the Longshanks mice reveals genomic signatures of selection”). However, it is not clear from the text or the supplement which other models (e.g. few genes of major effect, or a combination of polygenic and major effect loci), besides the infinitesimal model, were tested. The simulations regarding selection coefficients and LD are clear, but I couldn't find any other model regarding the general architecture of the trait.

3) The paper highlights the importance of standing genetic variation in rapid adaptive responses, and the validation of the candidate SNPs exemplifies this beautifully. However, nothing is mentioned about de novo mutations and the role they could play. A discussion about the relative importance of these two factors, or at least the mention of how many de novo SNPs were found in F17 is warranted. I acknowledge that it is not possible to test whether de novo SNPs increased in frequency in the population given that only F0 and F17 were sequenced.

4) The results are very interesting but poorly discussed. The Discussion section is focused more on highlighting the results than in actually discussing the findings in the context of previous selection studies. I would like to see the data discussed in the framework of what we expect or have seen before regarding e.g. the role of standing variation vs de novo mutations, polygenic vs major-loci signatures of selection, coding vs non-coding changes, etc. These are current (and old) debates in evolutionary biology that will benefit a lot from the results of this paper. Also, the fact that only two loci are replicated between LS1 and LS2, and that everything else, including other major-effect loci are unique to each line is mentioned in pass in the results but never discussed.

5) Related to the above point. In the Discussion section the effect size of the major loci is mentioned to be 10%, is this a high, low? Expected? There's no discussion about the effect sizes in terms of what other studies might have found. If 10% is the effect of a single locus how much is attributed to the polygenic component? Is there something else left to be explained? If modelling uses only standing-variation, is there a role for de novo mutations?

*Reviewer #2:*

I think this is really beautiful work at several levels, and in particular the very careful analysis of expectations under polygeny is exceptional. The clear demonstration that polygenic adaptation would leave almost no signature in data of this type is terrific.

My one substantive concern is that the authors conclude that polygeny played an important role in the evolution of their focal trait (e.g., line 188-190, and in the abstract), but it's not actually clear to me how much evidence there is to support that conclusion. Most of the genome is similar to their polygenic null expectation, but not all: they detected eight genome-wide significant loci, and they show an excess of signal below that threshold (e.g., Figure 2—figure supplement 2, maybe Figure 3A, maybe Figure 6A). Further, there's not obviously a test that compares their results to a qtls-but-no-polygenes null. I'd like to see more explicit accounting of *how much* variation is explained by polygenic effects.

A second very modest concern has to do with the circular mating scheme, which is mentioned only deep in the supplementary methods. I'd like to see this explained more. As I understand it, each mouse mates only once, yielding a single brood. In that case, circular mating involves lots of obligate first-cousin matings, preserving F-sub-B at the expense of F-sub-W. The higher frequency of autozygosity under this scheme will slightly reduce effective recombination relative to the genetic map. I *think* that the simulations account for this as they use the actual pedigree, but that should be stated more clearly. The increased autozygosity also changes the potential role of dominance in the estimation of the selection coefficient for Nkx3-2, although I'm sure it's a very slight effect.

*Reviewer #3:*

In this paper, Castro et al., analyze genomic data from a selection of mice. Two replicate populations were selected for long legs for 20 generations and compared to a single control population. There was a moderate response to selection, parallel in both replicates at the phenotype scale. Castro et al., follow-up on one major locus with functional genetic experiments. These provide evidence for the involvement of the gene Nkx3-2 in the response to selection. The dissection of the QTL down to 6 candidate quantitative trait nucleotides (QTNs) is a noteworthy advance for this kind of work in mice.

As a model for nature, the results apply only to very small populations. Perhaps this is the intention as these would be under greatest conservation threat. The result that parallel evolution was limited to loci of largest effect is entirely expected given the small population size. As population size increases, selection would become increasingly deterministic and parallel change more likely.

A strong feature of the experiment is that the full pedigree of the population is known. This allows the simulation modeling based directly on the pedigree of the population. However, several things here need to be further developed or clarified. Regarding the pedigree, why not use this to test for a change in the additive variance over the course of the experiment. The most important practical effect of having large effect loci for quantitative traits is that additive (co)variances change on the same time scale as means with selection. Figure 1B,C does suggest a slowing of response in the latter half of experiment. Can this be attributed to a reduction in the additive variance? If estimated, can the change be explained in terms of the allele frequency shifts at the 'major' loci?

Related to this, the text in subsection “Linking molecular mechanisms to evolutionary consequence*”* suggests that 1569 mice were genotyped at the major locus. The authors look at allele frequency change. However, the paper suggests that they also have individual phenotypes for each of these same mice. If so, the authors can estimate QTL effect on phenotype directly. An estimate for the average effect could be coupled with the known strength of selection on the phenotype to produce an independent prediction for allele frequency change. This procedure would not be associated with the "winner's curse" problem (effect overestimation of outliers) that the authors correctly note about the cumulative δ p estimate.

# Reply: Author response

**DOI:** 10.7554/eLife.42014.049

Essential revisions:

[…] First, it must be made clearer which data are new, and which were previously published (presumably in Marchini et al., 2014).

We agree that the original text as submitted was overly brief in discussing how this study differs from the foundational study that introduced the Longshanks experiment (Marchini et al., 2014). This was due to the need to fully convey essential details of the experiment while keeping the text as succinct as possible. While we acknowledge the necessary overlap in some of the underlying data, the text does benefit from a coherent presentation for maximal accessibility, without repeating previously published results. We therefore believe that we are justified in summarizing some basic overall features of the selection response at the trait level to the current generation 20. Beyond these basic features, we refer the readers to Marchini et al., 2014 for the analyses on trait correlations and trait response to selection.

We do agree, however, that we can improve the presentation here and make clear the novel aspects in the current study. We have therefore revised the text to read:

Introduction, “Previously, Marchini et al. investigated how selection was able to overcome correlation between tibia length and body mass and produced independent changes in tibia length during the first 14 generations of the Longshanks experiment (*13*). Importantly, that study focused on the phenotypes and inferred genetic correlations indirectly using the pedigree. The current genomic analysis was initiated when the on-going experiment reached generation 17 and extends the previous study by integrating both phenotypic *and* genetic aspects of the Longshanks experiment.”;

and

Introduction: [Description of breeding and phenotyping scheme]… “[only details essential to understanding our analysis are summarized here. See (Marchini et al., 2014) for a detailed description of the breeding scheme]”.

Second, although the polygenic analysis is an important part of this paper, we noted that no formal comparison with a simpler QTL model is included. How much of the selection response was due to each? Either add some analysis along these (perhaps test how adding more loci improves cross-replicate prediction?), or tone down the language.

The reviewers have rightly pointed out that we have not included a full and formal comparison between our infinitesimal model with linkage and alternative models with a few major QTLs. We agree that this is in principle a very interesting analysis and have discussed several ways to do it during the preparation of the manuscript. We have however concluded that embedding the effects of many QTLs within an infinitesimal model with variable genetic variance along the genome is unfortunately not at all easy – even without additional complications, such as modelling shared vs. unique loci in the two lines. That was why we only describe the discrete loci as those not captured by the infinitesimal model and formally estimated the selection coefficient and contribution to trait response to the experimentally validated top locus *Nkx3-2*.

In describing our results, we are also mindful of the power and strength of each analysis and what we could reasonably conclude from them. We hope our readers would note that at the end of each Results section, we have updated our summaries based on the accumulation of increasingly strong data points supporting the role of major loci, to finally modifying our model to incorporate the *Nkx3-2* locus (see Results section). The reviewers are no doubt equally aware that inverting our modeling scheme by (over)fitting individual loci first would not be valid. Given the rather classical phenotypic selection response observed, it is our view that we have chosen the most appropriate treatment of the data.

Our analysis of a discrete allele embedded in a polygenic background (Figure 5—figure supplement 1C) does show that a single-locus diffusion approximation captures the variability seen in simulations that also include the infinitesimal background. This gives us confidence in the limits we have set on the estimated effect of the *Nkx3-2* locus between 3.6% and 15.5% of the total response. These broad limits make it clear that we cannot give an accurate estimate of the response explained by the loci that were significant within LS1 and LS2: understanding how to properly attribute the response to discrete loci versus an infinitesimal background is a considerable challenge that we leave for the future.

As suggested, we have opted instead to tone down the language and have revised the text to read: Subsection “Sequencing the Longshanks mice reveals genomic signatures of selection*”*: “Thus, we conclude that the although genetic basis of the selection response in the Longshanks experiment may be largely polygenic, evidence strongly suggests discrete loci with major effect, even when each line is considered separately.”

Reviewer #1:

[…] 1) This study is based on the selection experiment by Marchini et al., (2014). However, in the text it is never clear which data are new and which are from Marchini, 2014 (e.g. there they explore mice up to the thirteenth generation). The only hint is in the Introduction. The way the text is currently written suggests the Lonshank experiment is a new contribution of this manuscript.

See our response to Essential revisions #1 above.

2) The authors claim that an infinitesimal model with linkage "best fits the observed data" (subsection “Sequencing the Longshanks mice reveals genomic signatures of selection”). However, it is not clear from the text or the supplement which other models (e.g. few genes of major effect, or a combination of polygenic and major effect loci), besides the infinitesimal model, were tested. The simulations regarding selection coefficients and LD are clear, but I couldn't find any other model regarding the general architecture of the trait.

We agree that the wording here was ambiguous. As stated in our response to Essential revision #2 above, we have opted not to compare other models. What we were referring to was indeed the choice of the LD and amount of selection, with the results already shown in Figure 1—figure supplement 2E. We have therefore revised the text to read:

Subsection “Sequencing the Longshanks mice reveals genomic signatures of selection”: “infinitesimal selection model with strong LD amongst marker SNPs performed better than moderate LD or no LD (Figure 1—figure supplement 2E).”

3) The paper highlights the importance of standing genetic variation in rapid adaptive responses, and the validation of the candidate SNPs exemplifies this beautifully. However, nothing is mentioned about de novo mutations and the role they could play. A discussion about the relative importance of these two factors, or at least the mention of how many de novo SNPs were found in F17 is warranted. I acknowledge that it is not possible to test whether de novo SNPs increased in frequency in the population given that only F0 and F17 were sequenced.

During early phases of analysis, we have indeed made an attempt to discover de novomutations by summarizing SNPs found in F17 that were absent in F0. However, we quickly realized that this simple approach gave >10,000 SNPs, a rate far in excess of known SNP mutation rates. The most likely explanation beyond sequencing error would be missing F0 founder individuals that we were unable to recover from the archive. It soon became clear that in order to generate reliable calls for seeding our simulations with founder haplotypes, a decision was made to be conservative and only call known alleles. For that reason, we did not want to report an inflated de novoSNP count to create unnecessary complications.

More generally, the contribution of new mutations to additive genetic variance is surprisingly high, with *V_m_*=0.001-0.01*V_e_* for a variety of traits and organisms (Lynch and Walsh, 1998). Nevertheless, such rates are not enough to contribute significantly to selection response within 20 generations (Hill, 1982; Weber and Diggins,1990). In our experiment, new mutations could arise in one or other selected line, and if of sufficiently large effect, could contribute to the selection response. However, their effects on the final SNP frequencies would be indistinguishable from that of a rare standing variant that was established in just one selected line.

In the future, we do intend to analyze the sequencing of intermediate generations, which of course could detect input from new mutations. However, the well-established estimates of *V_m_* give us confidence that our focus on standing genetic variants is appropriate for the current dataset, which focuses on F0 and F17 generations.

4) The results are very interesting but poorly discussed. The Discussion section is focused more on highlighting the results than in actually discussing the findings in the context of previous selection studies. I would like to see the data discussed in the framework of what we expect or have seen before regarding e.g. the role of standing variation vs de novo mutations, polygenic vs major-loci signatures of selection, coding vs non-coding changes, etc. These are current (and old) debates in evolutionary biology that will benefit a lot from the results of this paper. Also, the fact that only two loci are replicated between LS1 and LS2, and that everything else, including other major-effect loci are unique to each line is mentioned in pass in the results but never discussed.

We agree with the reviewers. The Discussion section in our submitted MS was indeed far too short. We have now expanded the Discussion section and have put our findings into context. We believe that this current revision addresses where we extend previous work and also highlights the necessary limitations in our study due to sample size, replicate numbers and length of selection. We believe this expanded Discussion section is a balanced summary of the state of the field and can highlight areas in need of attention in future theoretical and empirical studies.

5) Related to the above point. In the Discussion section the effect size of the major loci is mentioned to be 10%, is this a high, low? Expected? There's no discussion about the effect sizes in terms of what other studies might have found. If 10% is the effect of a single locus how much is attributed to the polygenic component? Is there something else left to be explained? If modelling uses only standing-variation, is there a role for de novo mutations?

See our response to Essential revisions #2 above. Essentially, we have presented our best attempt to summarize the contribution, given the inherent uncertainties and limitations in any such experiments. Put simply, our estimates after adding additional loci will become increasingly uncertain, with wide confidence bounds. We can briefly illustrate our point below.

For the top locus, our estimate was 9.4% (bounds: 3.6-15.5%). There are in total 3 loci significant in LS1, 7 in LS2, 2 of these being shared; the selection coefficients sum up to 0.61 in LS1, 1.22 in LS2; thus, extrapolating from the previous calculation, the significant loci would in principle account for 22%, 44% of the response in LS1, LS2 respectively. However, given the wide confidence intervals, the fraction of response explained might range from <10% in LS1, to >70% in LS2. These rough estimates just emphasize the uncertainties that accumulate as one considers more (and weaker) candidate loci. A proper calculation of the fraction of response explained would be decidedly non-trivial.

Reviewer #2:

[…] My one substantive concern is that the authors conclude that polygeny played an important role in the evolution of their focal trait (e.g., line 188-190, and in the abstract), but it's not actually clear to me how much evidence there is to support that conclusion. Most of the genome is similar to their polygenic null expectation, but not all: they detected eight genome-wide significant loci, and they show an excess of signal below that threshold (e.g., Figure 2—figure supplement 2, maybe Figure 3A, maybe Figure 6A). Further, there's not obviously a test that compares their results to a qtls-but-no-polygenes null. I'd like to see more explicit accounting of *how much* variation is explained by polygenic effects.

See our response to Essential revisions #1 above.

A second very modest concern has to do with the circular mating scheme, which is mentioned only deep in the supplementary methods. I'd like to see this explained more. As I understand it, each mouse mates only once, yielding a single brood. In that case, circular mating involves lots of obligate first-cousin matings, preserving F-sub-B at the expense of F-sub-W. The higher frequency of autozygosity under this scheme will slightly reduce effective recombination relative to the genetic map. I *think* that the simulations account for this as they use the actual pedigree, but that should be stated more clearly. The increased autozygosity also changes the potential role of dominance in the estimation of the selection coefficient for Nkx3-2, although I'm sure it's a very slight effect.

The mating system was chosen to minimise inbreeding, and indeed, the effective size of *N_e_*~45 was ~50% greater than the actual number of mice. As the reviewer points out, the actual heterozygosity will differ from that in a random mating population with the same *N_e_*, which in turn will alter the effective recombination rate. However, the difference is very slight, and in any case, the simulations used the actual pedigree, and so include this effect. Moreover, we compared simulated allele frequency distributions with the diffusion approximation (Figure 1—figure supplement 2D) and saw no detectable difference.

Reviewer #3:

In this paper, Castro et al., analyze genomic data from a selection of mice. Two replicate populations were selected for long legs for 20 generations and compared to a single control population. There was a moderate response to selection, parallel in both replicates at the phenotype scale. Castro et al., follow-up on one major locus with functional genetic experiments. These provide evidence for the involvement of the gene Nkx3-2 in the response to selection. The dissection of the QTL down to 6 candidate quantitative trait nucleotides (QTNs) is a noteworthy advance for this kind of work in mice.

As a model for nature, the results apply only to very small populations. Perhaps this is the intention as these would be under greatest conservation threat. The result that parallel evolution was limited to loci of largest effect is entirely expected given the small population size. As population size increases, selection would become increasingly deterministic and parallel change more likely.

We agree and now have added language to highlight this aspect of our study, which may bias the results against parallelism.

Subsection “Sequencing the Longshanks mice reveals genomic signatures of selection”: “However, one should bear in mind the very many genetic paths to increasing tibia length under an infinitesimal model, and that the effect of drift is expected to be very strong in these small populations. In larger populations, the shift in the balance from drift to selection should result in selection being able to favor increasingly subtle variants and thus produce a greater proportion of parallel loci. However, we expect the trend of parallelism being enriched among the top loci to hold."

Materials and methods section: With increasing population size, selection would be better able to detect variants with more subtle effects. This would turn lower the threshold beyond which the selection advantage of an allele would become deterministic, i.e., exhibit parallelism.”

A strong feature of the experiment is that the full pedigree of the population is known. This allows the simulation modeling based directly on the pedigree of the population. However, several things here need to be further developed or clarified. Regarding the pedigree, why not use this to test for a change in the additive variance over the course of the experiment. The most important practical effect of having large effect loci for quantitative traits is that additive (co)variances change on the same time scale as means with selection. Figure 1B,C does suggest a slowing of response in the latter half of experiment. Can this be attributed to a reduction in the additive variance? If estimated, can the change be explained in terms of the allele frequency shifts at the 'major' loci?

We have added estimates for additive genetic variance (as narrow-sense heritability) in Figure 1—figure supplement 1D and see no reduction over 20 generations in either selected line. Note that since the reduction in heterozygosity over 17 generations is modest, and uncertainties in estimating additive genetic variance are high, this is not surprising.

Related to this, the text in subsection “Linking molecular mechanisms to evolutionary consequence” suggests that 1569 mice were genotyped at the major locus. The authors look at allele frequency change. However, the paper suggests that they also have individual phenotypes for each of these same mice. If so, the authors can estimate QTL effect on phenotype directly. An estimate for the average effect could be coupled with the known strength of selection on the phenotype to produce an independent prediction for allele frequency change. This procedure would not be associated with the "winner's curse" problem (effect overestimation of outliers) that the authors correctly note about the cumulative δ p estimate.

We thank the reviewer for this suggestion. We used a linear mixed “animal model” to estimate the effect of the enhancer N3 (of the major locus in Nkx3-2) on the composite selected trait *ln*(*TB*^-0.57^), see subsection “Simulating selection response: infinitesimal model with linkage*”* and Figure 1—figure supplement 2A. The model was:

*V*_P_ = fixed effects + *V*_A_ + *V*_R_

where:

fixed effects = sex, generation, litter size (i.e., number of siblings in family), genotype at N3 (0, 1, or 2 copies of F17 allele), and replicate line

*V*_A_ = additive genetic variance

*V*_R_ = residual variance

We found a small but significant effect of the genotype at enhancer N3 on the composite trait (mean effect = 0.003568; 95% CI: 0.0006828 – 0.006369; *P*=0.0171). Given the same body mass *B*, the mean effect corresponds to 0.36% increase in tibia length per copy of the F17 allele, or ~1% of the variance in tibia length at generation F01. The observed increase of this allele from ~0.18 to 0.91, averaged over the two lines, implies that it accounts for ~4% of the total selection response. This is within the confidence limits in the main text, based on the change in SNP frequency (3.6%-15.5%) – and note that the latter may be biased upwards by ascertainment. However, the exact effect of the allele is difficult to pinpoint in any given generation or population due the nature of the composite trait and change in variance in the composite trait over generations.
